# Optical Activity and Spin Polarization: The Surface Effect

**DOI:** 10.1021/jacs.2c10456

**Published:** 2023-02-11

**Authors:** Tzuriel S. Metzger, Harikrishna Batchu, Anil Kumar, Daniil A. Fedotov, Naama Goren, Deb Kumar Bhowmick, Israa Shioukhi, Shira Yochelis, Igor Schapiro, Ron Naaman, Ori Gidron, Yossi Paltiel

**Affiliations:** † Department of Applied Physics and Center for Nanoscience and Nanotechnology, The Hebrew University, Jerusalem 9190401, Israel; ‡ Institute of Chemistry and Center for Nanoscience and Nanotechnology, The Hebrew University, Jerusalem 9190401, Israel; § Department of Chemical and Biological Physics, Weizmann Institute, Rehovot 76100, Israel

## Abstract

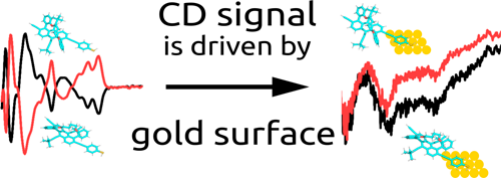

Chirality (‘handedness’) is a property that underlies a broad variety of phenomena in nature. Chiral molecules appear in two forms, and each is a mirror image of the other, the two enantiomers. The chirality of molecules is associated with their optical activity, and circular dichroism is commonly applied to identify the handedness of chiral molecules. Recently, the chiral induced spin selectivity (CISS) effect was established, according to which transfer of electrons within chiral molecules depends on the electron’s spin. Which spin is preferred depends on the handedness of the chiral molecule and the direction of motion of the electron. Several experiments in the past indicated that there may be a relation between the optical activity of the molecules and their spin selectivity. Here, we show that for a molecule containing several stereogenic axes, when adsorbed on a metal substrate, the peaks in the CD spectra have the same signs for the two enantiomers. This is not the case when the molecules are adsorbed on a nonmetallic substrate or dissolved in solution. Quantum chemical simulations are able to explain the change in the CD spectra upon adsorption of the molecules on conductive and nonconductive surfaces. Surprisingly, the CISS properties are similar for the two enantiomers when adsorbed on the metal substrate, while when the molecules are adsorbed on nonmetallic surface, the preferred spin depends on the molecule handedness. This correlation between the optical activity and the CISS effect indicates that the CISS effect relates to the global polarizability of the molecule.

## Introduction

The description of chirality is usually dichotomic, if particular object is chiral, its mirror image exhibits the opposite enantiomer.^1^ The degree of chirality can be defined based on a structural geometry difference^2,3^ or on the degree of optical activity at a given wavelength. The two definitions, the space symmetry structural difference and the degree of optical activity, are not always similar. Thus, it is very difficult to comprehensively measure chirality and define the chirality magnitude. Chirality may also be influenced by the environment and the substrate for adsorbed molecules.^4,5^

Since the beginning of this millennium, another interesting phenomenon related to chiral materials was discovered. It is the chiral induced spin selectivity (CISS) effect.^6^ The effect results in the dependence of electrons’ motion through a chiral system on their spin. Which spin is preferred depends on the handedness of the molecule and on the direction of motion of the electron.^7−9^ Hence, in principle, the CISS effect can serve as a means to measure the extent of chirality. As opposed to CISS, circular dichroism (CD) is a well-established and fundamentally understood phenomenon.^10,11^ It depends on the scalar product between the electric and magnetic transition dipoles of the material.^11,12^ Hence, it can be calculated with relatively good accuracy.^13^ It is important to note, however, that while CD spectra are usually taken for molecule in solution, CISS measurements are performed when the molecules are typically attached to electrodes or adsorbed on surfaces.

Still, qualitative correlation was found between the sign and magnitude of the first, the lowest energy, Compton peak in the CD spectra and the sign and magnitude of the spin polarization resulting from the CISS effect.^14,15^ For example, in π-conjugated materials, the correlation between the CD and spin filtration properties has been shown for supramolecular nanofibers.^16^ In this study, the authors demonstrated a control over the spin selectivity by changing the ratio between building blocks and conformational modifications, a phenomenon called “sergeants and soldiers”.^17^

So far, no attempt was made, to the best of our knowledge, to observe the CISS effect in complex chiral structures where several stereogenic elements exist and to probe how the effect depends on the substrate. It is shown here that despite the dramatic changes that may occur in the CD spectra of chiral molecules adsorbed on metal surface, the CISS effect still follows these modified spectra. The results shed new light on the possible mechanism of the CISS effect and on its relation to the optical activity of the chiral material. The result also points to the possible dramatic change in optical activity of chiral molecules when adsorbed on different surfaces.

## Results and Discussion

**Figure 1:**
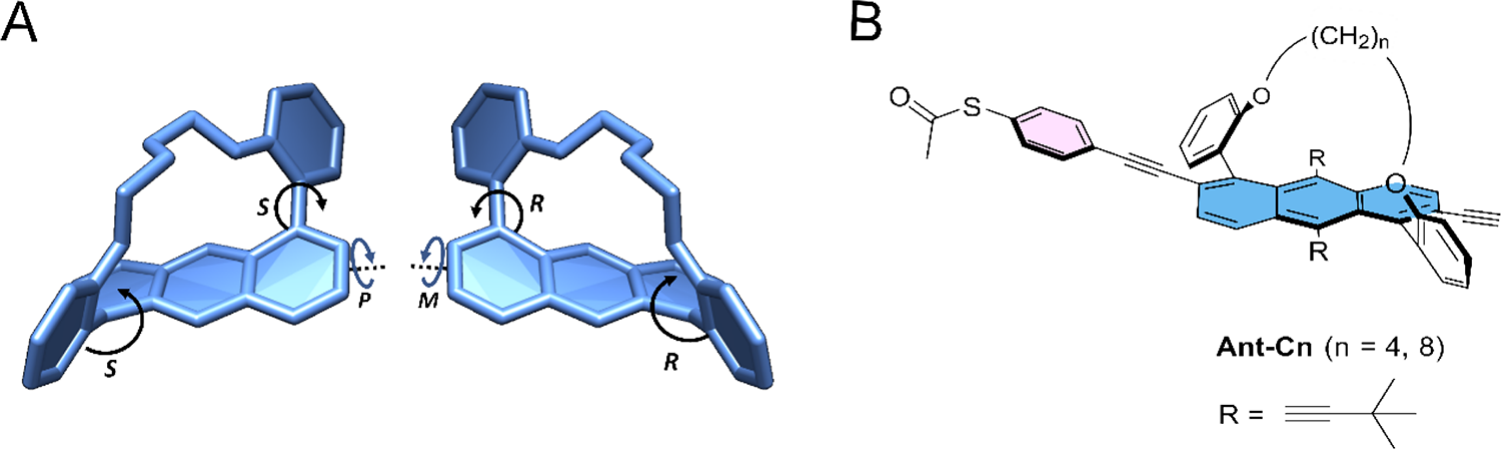
(A) Representative stereogenic axes of the Ant-Cn backbone. (B) Structure of Ant-Cn, with *n* = 4, 8 (butyl or octyl bridges, respectively). The alkyl bridge between two ether groups controls the degree of end-to-end twist.

A family of twisted anthracene molecules, Ant-Cn, in which the tether length controls the degree of twisting was recently introduced.^18,19^ The core of these molecules is depicted in Figure 1A. Ant-C8 consisting of an *n*-octyl tether is twisted by 20°, and Ant-C4, with the shorter *n*-butyl tether, is twisted by 40°. This *C*_2_ symmetric scaffold consists of three stereogenic axes: two axially stereogenic axes between the phenyls and anthracene as well as the twisted anthracene (Figure 1A).^18^ The degree of chirality depends on the backbone twist, with the shorter tether resulting in a larger backbone twisting and a stronger CD activity. We have shown previously that the chiroptical activity depends linearly on the degree of backbone twisting.^20^ Full description on synthesis and characterization including NMR, UV, MALDI-TOFMS, and XPS spectra is available in detail in SI sections S1–S4 and Figures S1–S22.

To allow the adsorption of these molecules on the surface, we have functionalized one end of the anthracene moiety with a thioester group, using Sonogashira coupling of tethered twistacenes with terminal alkyne moieties with 4-iodophenyl thioacetate to result in Ant-C4 and Ant-C8 (Figure 1B, see section S1 in the SI for synthetic details).^21^ Separation with preparative chiral HPLC resulted in enantiopure *M* and *P* enantiomers for each Ant-Cn.

**Figure 2:**
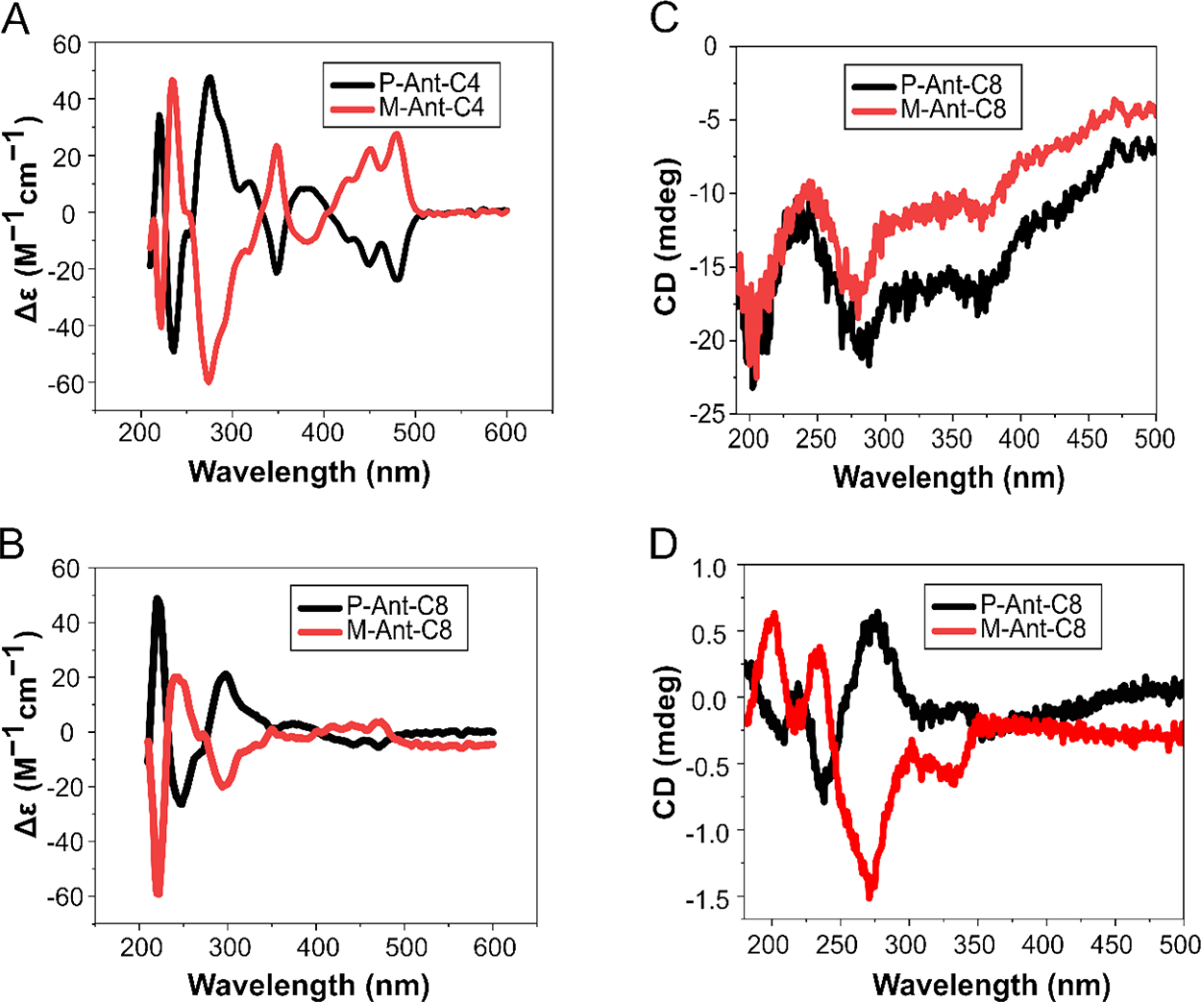
CD spectra of enantiopure (A) Ant-C4 and (B) Ant-C8 measured in acetonitrile. (C) CD spectra of Ant-C8 measured when the molecules are adsorbed on gold or (D) when the molecules are adsorbed on quartz. The CD signal was obtained by subtracting the signal from the clean substrate from the total signal. Each sample was measured several times, with light coming at normal to the substrate.

The CD spectra of the enantiopure molecules were measured in acetonitrile solution. As expected, the *M* and *P* enantiomers show mirror image spectra, as presented in Figure 2A,B. However, when the molecules are adsorbed on a gold substrate, the CD spectra of the two enantiomers are similar in shape, although the signal of the *P* enantiomer is stronger by about 20% (Figure 2C). When the molecules are adsorbed on nonconducting substrate, like quartz, the CD spectra are very similar to that observed in solution (Figure 2D). Measuring the CD of a monolayer was done using many substrates all perfectly aligned. For more details on preparation of self-assembled monolayers for CD measurements, see SI section S5.

**Figure 3:**
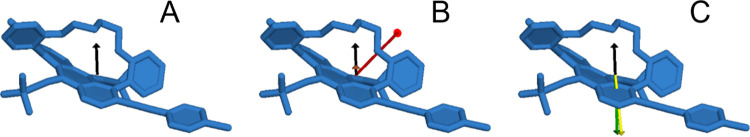
*P* enantiomer of Ant-C8 and the simulated transition dipole and magnetic moments. (A) Normal vector (black) of the gold surface. The normal vector is used for projection of the transition dipole and magnetic moments due to the presence of the gold surface. (B) Original (red) and projected (brown) transition dipole moments (TDM and projected TDM). The angle between TDM and the normal vector of the plane is ∼87°, which explains the small size of the projected TDM. For clarity, the length of both vectors was scaled by a factor of 6. (C) Original (yellow) and projected (green) transition magnetic moments (TMM and projected TMM). The angle between TMM and the normal vector of the plane is ∼173°, thus explaining the small change in length. For visualization purposes, the length of both vectors was scaled by a factor of 10.

To understand the change in the CD spectra upon adsorption of Ant-C8, we have performed quantum chemical simulations (see details in SI section S7 and Tables S25–S28). Due to the prohibitive computational cost, the gold surface was not explicitly included in the simulation. Calculating an isolated Ant-C8 molecule is justified by the fact that the CD spectrum is determined by the configuration of the organic molecule. However, we assume that the anthracene unit of Ant-Cn is oriented parallel to the surface. Due to the twist of the anthracene, we have determined an average plane to define the normal vector of the surface (Figure 3a). The transition dipole (μ_e_) and magnetic moments (μ_m_) for the first excited state are shown in Figure 3b,c, respectively. The intensity of the CD spectrum is proportional to μ_e_·μ_m_.^11^ The results indicate that the electric transition dipole moment for the first electronic excitation is in the plane of the anthracene moiety, while the magnetic transition dipole moment is out-of-plane. Considering the orientation of the surface, we have visualized the cancelation of the transition dipole moment and transition magnetic moment components, which are parallel to the gold surface (Figure 3b,c). If the anthracene unit of Ant-C8 is adsorbed parallel to the gold surface, the electric transition dipole moments would be reduced by the same extent for both enantiomers. Such an adsorption would result in different rotatory strengths. However, due to the twist of the anthracene moiety, it is more likely that one ring of the anthracene unit is adsorbed to the surface. Hence, the electric transition dipole moment is not parallel to the surface. Therefore, the same sign of the rotatory strength can only be explained if the sign is changing for one of the enantiomers because it is not parallel to the surface (see Figure 3).

**Figure 4:**
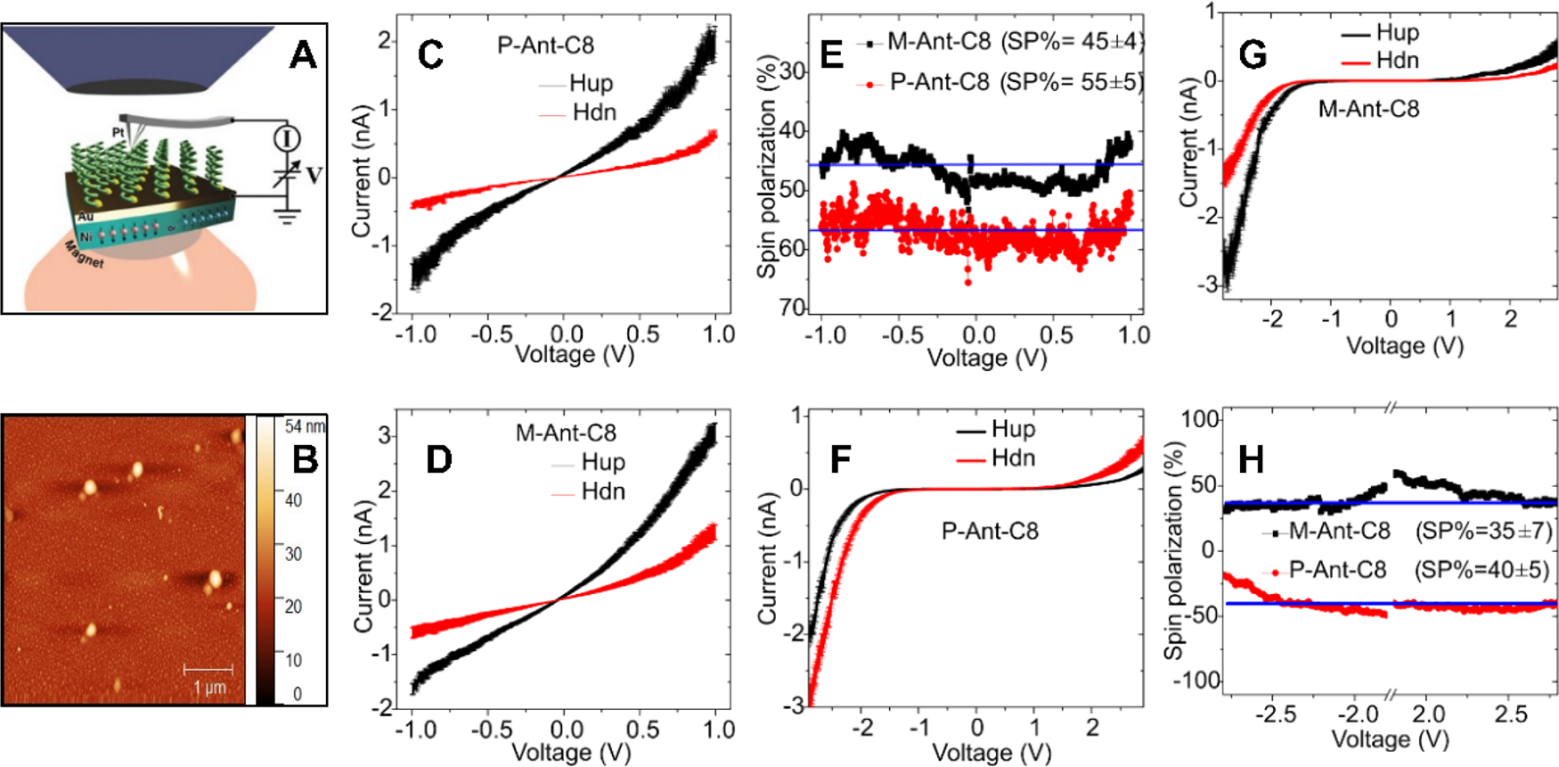
Spin-dependent current through the enantiomers measured by mc-AFM. (A) Experimental setup in the case of a gold substrate. The 5 nm gold layer is deposited on a nickel layer that can be magnetized with the magnetic field pointing toward the molecules (up) or away from the molecule (dn) (adapted with permission from ref (28), copyright 2020 John Wiley and Sons). (B) AFM surface image. (C, D) Current versus voltage curves for molecules adsorbed on gold. For both enantiomers, the highest current is observed for the magnet is pointing up. (E) Spin polarization, *P*, calculated from curves B and C. *P* = (*I*_up_ – *I*_dn_)/(*I*_up_ + *I*_dn_), where *I*_up_ and *I*_dn_ are the current measured when the magnet is pointing up or down, respectively. In this case, the spin polarization has the same sign for both enantiomers. When the molecules are adsorbed on ITO, the tip of the AFM is magnetized. (F, G) Current versus voltage signal for the tip magnetized up or down (black and red curves, respectively) for the two different enantiomers adsorbed on ITO. (H) Spin polarization obtained in the case of enantiomers adsorbed on ITO. The spin polarization has opposite signs for the two enantiomers.

The spin-selective transport through the molecules was measured using the magnetic contact atomic force microscopy (mc-AFM) setup (Figure 4A).^9,22^ For the molecules adsorbed on metal, we used a setup in which the substrate is gold-coated nickel that was magnetized perpendicular to the surface with the north pole pointing toward the adsorbed layer (up) or away from it (dn). The spin of the electrons injected from the substrate into the molecules or vice versa depends on the direction of magnetization. Indeed, as shown in Figure 4C,D, when the substrate is magnetized with the magnetic field pointing up, the current is higher for both enantiomers.

Hence, the spin polarization (Figure 4D) has the same sign for both enantiomers (Figure 4E), although the polarization is higher for the *M* enantiomer. The spin polarization, *P*, is defined as *P* = (*I*_up_ – *I*_dn_)/(*I*_up_ + *I*_dn_), where *I*_up_ and *I*_dn_ are the current measured when the magnet is pointing up or down, respectively. The differences between the *M* and *P* enantiomers measured both in the CD signal and similarly in spin polarization could be ascribed to dependence of the CD on chiral alignment,^23,24^ and a similar trend is expected for analysis of spin polarization.^25^

When the molecules are adsorbed on ITO, the tip of the AFM is magnetized instead of the substrate. The current versus voltage signal for the tip magnetized up or down for the two different enantiomers indicates a different spin preference for each enantiomer (Figure 4F,G). Indeed, the spin polarization obtained in the case of enantiomers adsorbed on ITO has opposite signs for the two enantiomers (Figure 4H). More details on the mc-AFM measurements are in SI section S6 and Figures S23 and S24.

**Figure 5:**
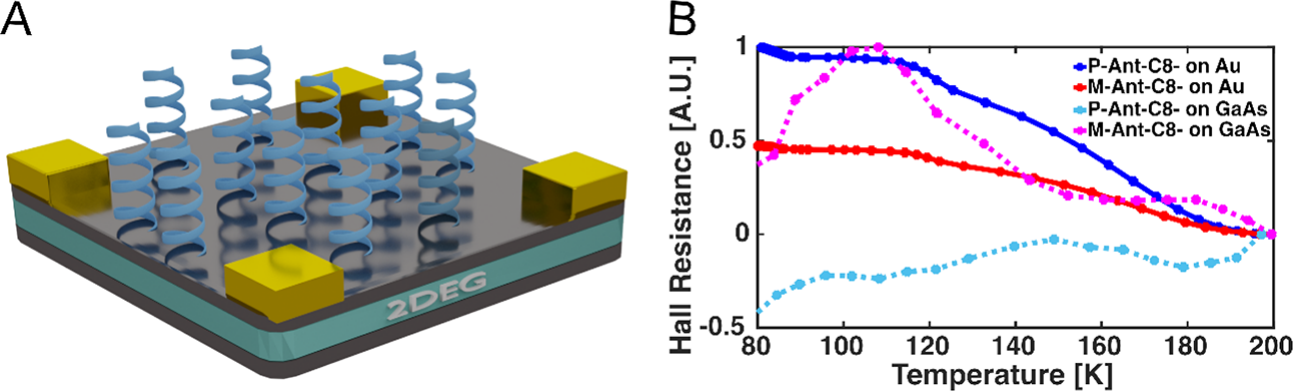
Measurements of spin polarization by Hall devices. (A) Scheme of the Hall device. (B) Temperature-dependent signal of the Hall device when the molecules are adsorbed on gold-coated surface (solid red and blue lines) or on the GaAs substrate (dotted pink and light blue lines). It is important to note that the Hall response combines anomalous responses and standard linear responses; therefore, it is hard to quantify the ratio between the responses.

It was shown previously that the CISS effect can be validated by Hall effect measurements.^14,15^ The substrate in this case contain a two-dimensional electron gas (2DEG) GaAs/AlGaAs device. This was done to have a sensitive substrate for Hall measurements, as described in previous works.^14,15^ In one configuration of the experiment, the Ant-Cn molecules were adsorbed on a thin gold film that coated the GaAs layer, while in the other configuration, the molecules were adsorbed directly on the nonmetallic GaAs substrate. The device is shown schematically in Figure 5A. Hall resistance was measured in a van der Pauw configuration as a function of temperature, and each device was measured before and after adsorption.

The temperature-dependent studies (Figure 5B) are consistent with the spin transport measurements (Figure 4) and indicate that while in the case of the gold substrate, the sign of the Hall signal is the same for the two enantiomers (solid lines), it is the opposite when the enantiomers are adsorbed on the nonmetallic substrate (doted lines). It is important to take into account that the changes in the CD upon adsorption on surfaces may result from several effects and not only be due to the metallic properties of the substrate. These effects may include chiral alignment, contribution from linear dichroism, etc. However, the important conclusion from the work described here is that CISS and spin polarization are correlated with the circular dichroism signal and as a result must be related to many electron effects.

## Conclusions

In this work, it was shown that when a nonplanar chiral molecule, with several stereogenic axes, is adsorbed on a metal substrate, its CD spectrum may change dramatically to the extent that the two enantiomers have the same sign of the Compton peaks in the spectra. This phenomenon is explained by the canceling of the components of the electric dipole moments that are parallel to the metal surface. This canceling is of course a well know phenomenon; however, it is interesting that in some cases, it can lead to what seems to be “disappearance” of the mirror image in the CD spectra for the two enantiomers. Even more striking is the finding that the spin polarization, resulting from the CISS effect, follows the CD spectra. The loss of the mirror image picture in the CD spectra of the two enantiomers coincides with the two enantiomers becoming spin filters with the same preferred spin for the electron conduction through the molecules.

The results emphasize that the mechanism of the CISS effect must involve global properties of the molecules, like anisotropic polarizability. Such a property also defines the optical activity of the system. Hence, the correlation between optical activity and spin polarization is now well-established. This conclusion is consistent with several models presented recently^9,26^ for the CISS effect, and it explains why a single electron theoretical models failed in reproducing the experimental results.^27^

## Supporting Information Available

Synthesis, sample preparation, measurements methods, theoretical methodology, and characterization measurements of Ant-Cn including: NMR, UV, MALDI-TOFMS, and XPS spectra and additional spin-dependent transport measurements (PDF)
