# Supplementary material for: Optical Activity and Spin Polarization: The Surface Effect
Source: J Am Chem Soc. 2023 Feb 11;145(7):3972–7. doi: 10.1021/jacs.2c10456 (PMC11139380; doi:10.1021/jacs.2c10456)
Supplement: Supplementary file 1 — ja2c10456_si_001.pdf [file ja2c10456_si_001.pdf]

Supporting Information

Optical Activity and Spin Polarization - The Surface Effect

Tzuriel S. Metzger[1#], Harikrishna Batchu[2#], Anil Kumar[3#], Daniil A. Fedotov[2#], Naama Goren[1], Deb Kumar Bhowmick[3], Israa Shioukhi[2], Shira Yochelis[1], Igor Schapiro,[2] Ron Naaman,[3]* Ori Gidron[2]* and Yossi Paltiel[1]*

[1]Department of Applied Physics and Center for Nanoscience and Nanotechnology, The Hebrew University, Jerusalem 9190401, Israel

[2]Institute of Chemistry and Center for Nanoscience and Nanotechnology, The Hebrew University, Jerusalem 9190401, Israel

[3]Department of Chemical and Biological Physics, Weizmann Institute, Rehovot 76100, Israel

# T.S.M., H.B., A.K. and D.F. contributed equally to this paper

* Corresponding authors

Ron Naaman – ron.naaman@weizmann.ac.il

Ori Gidron – Ori.Gidron@mail.huji.ac.il

Yossi Paltiel – paltiel@mail.huji.ac.il

S1 General

Commercially available reagents and chemicals were used without further purification unless otherwise stated. Compound **1-C8, 1-C4** and **2** was synthesized according to a previous reports[1,2].

^{1}H and ^{13}C NMR spectra were recorded in solution on a Bruker-AVIII 400 MHz and 500 MHz spectrometers using tetramethylsilane (TMS) as the external standard. The spectra were recorded using chloroform-d as the solvent. Chemical shifts are expressed in δ units.

UV-vis absorption spectra were recorded with an Agilent Cary-5000 spectrophotometer. The spectra were measured using a quartz cuvette (1 cm) at 25 °C. The absorption wavelengths are reported in nm with the extinction coefficient ε ($M^{-1}cm^{-1}$) in brackets. Electronic Circular Dichroism (ECD) spectra were recorded on a MOS-500 spectrophotometer from BioLogic Science Instruments.

MALDI-TOFMS spectra were acquired using an MALDI-TOF/TOF autoflex speed mass spectrometer (Bruker Daltonilk GmbH, Bremen, Germany) equipped with a smartbeam-II solid-state laser (modified Nd:YAG laser) λ = 355 nm. The instrument was operated in positive ion, reflectron mode. The accelerating voltage was 21.0 kV. The delay time was 130 ns. Laser fluence were optimized for each sample. The laser was fired 5 at a frequency of 2 kHz and spectra were accumulated in multiples of 500 laser shots to achieve 1500 shots in total. Sample preparation: 2-[(2E)-3-(4-tert-Butylphenyl)-2-methylprop2- enylidene] malononitrile (DCTB) matrix solutions were made to a concentration of 20 mg/mL in dichloromethane (DCM). Sample solutions were made to an approximate concentration of 5 mg/mL in DCM. Sample and matrix solutions were premixed in ratio of 1:10 or 1:40 (v/v). A volume of 0.5 µL of this mixture was disposed on a MALDI steel target plate. After evaporation of the solvent, the target was inserted into the mass spectrometer.

S2 Synthesis

S2.1 Synthesis of the Ant-Thioester

S2.1.1 Scheme S1. Synthesis of ***P*****-Ant-*****C8***

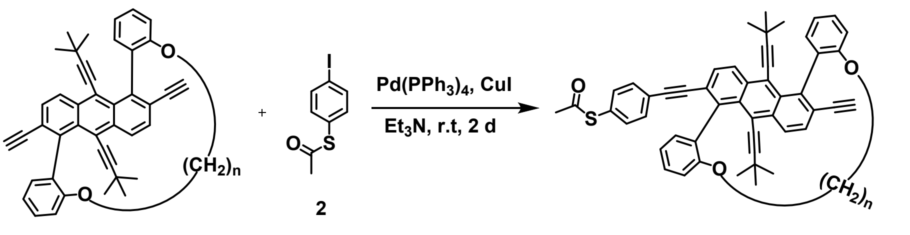

1-C8; n = 8
1-C4; n = 4

Ant-C8; n = 8, 41-44%
Ant-C4; n = 4, 36-40%

General method for synthesis of Ant-Thioester

Synthesis of ***P*-Ant-C8**

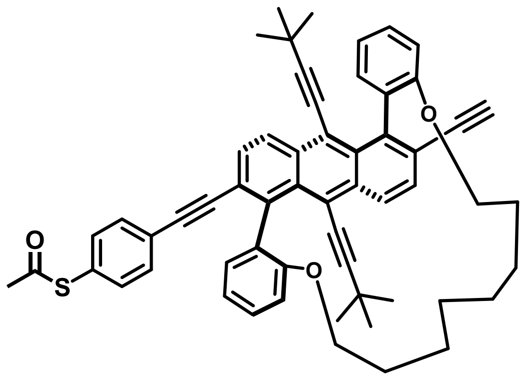

To a two necked RB flask kept under positive argon flow were added ***P*-1-C8** (44 mg, 0.065 mmol), (4-iodophenyl)ethanethioate (22 mg, 0.078 mmol), $Pd(PPh_3)_4$ (3.8 mg, 0.003 mmol) and CuI (1.2 mg, 0.006 mmol). Triethylamine (6 mL) was purged with argon for 5 min and then added to the previously mixed reagents and the reaction mixture was stirred at room temperature for 2 days. The solvent was evaporated and the reaction mixture was directly loaded on a silica gel column and eluted with EtOAc/hexane (1:5) to afford ***P*-Ant-C8** as a yellow solid (17.3 mg, yield = 41.3%) and recovered ***P*-1-C8** (10 mg, 22.6 %) .

^{1}H NMR (400 MHz, $CDCl_3$) δ 8.67 (dd, *J* = 8.1, 1.1 Hz, 2H), 7.61 (d, *J* = 9.0, 1H), 7.57 (d, *J* = 6.0, 1H), 7.46 – 7.38 (m, 4H), 7.30 – 7.27 (m, 2H), 7.15 – 7.11 (m, 2H), 7.10-7.05 (m, 2H), 6.95 (ddd, J = 8.3, 6.0, 1.1 Hz, 2H), 3.97-3.92 (m, 2H), 3.83-3.77 (m, 2H), 2.99 (s, 1H, -C≡C-H), 2.41 (s, 3H, -$SCOCH_3$), 1.32 (t, *J* = 6.0 Hz, 4H), 1.14 (d, *J* = 1.0 Hz, 18H), 0.66-0.53 (m, 8H) .

^{13}C NMR (100 MHz, Chloroform-*d*) δ 193.8, 158.2, 157.9, 141.7, 141.6, 134.9, 134.1, 132.6, 132.3, 131.8, 130.1, 129.9, 129.3, 129.2, 129.1, 128.3, 127.8, 127.8, 127.6, 125.1, 123.1, 122.2, 120.3, 120.2, 120.0, 119.9, 116.2, 116.1, 112.2, 112.0, 93.9, 92.6, 84.2, 81.8, 78.9, 78.8, 69.5, 31.0, 30.4, 29.7, 29.7, 29.4, 28.7, 27.1, 27.0.

MS (MALDI-TOF, m/z): calcd. for $C_{58}H_{54}O_3S$: 830.379; found 830.387.

UV-vis (CH_3CN): λ_{max} (ε) = 306 (50330), 329 (60432), 344 (76928), 397 (4287), 419 (7715), 444 (12822), 472 nm (15802 M^{-1} cm^{-1}).

Synthesis of ***M*-Ant-C8**

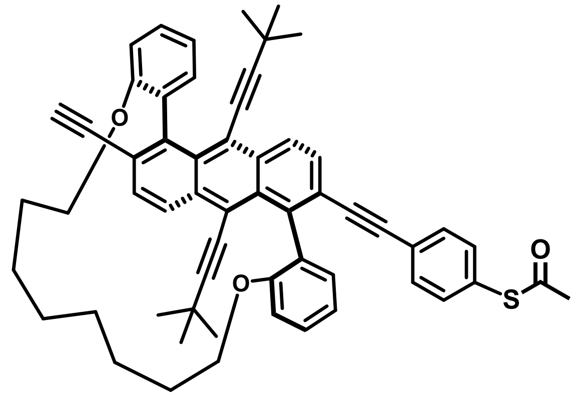

***M*-Ant-C8** was synthesized according to the same procedure as was used for ***P*-Ant-C8**, starting from ***M*-*1*-C8** (50 mg, 0.073 mmol), (4-iodophenyl)ethanethioate (24.7 mg, 0.089 mmol), $Pd(PPh_3)_4$ (4.2 mg, 0.004 mmol) and CuI (1.4 mg, 0.007 mmol). Triethylamine (6 mL). ***M*-Ant-C8** was isolated as a deep yellow solid (20 mg, yield = 43.7 %) and recovered ***M*-1-C8** (12 mg, 24.1 %).

^{1}H NMR (400 MHz, $CDCl_3$) δ 8.67 (dd, *J* = 8.1, 1.1 Hz, 2H), 7.61 (d, *J* = 8.0, 1H), 7.57 (d, *J* = 8.0, 1H), 7.46 – 7.35 (m, 4H), 7.30 – 7.27 (m, 2H), 7.15 – 7.11 (m, 2H), 7.09-7.05 (m, 2H), 6.95 (ddd, J = 8.3, 6.0, 1.1 Hz, 2H), 3.97-3.93 (m, 2H), 3.82-3.80 (m, 2H), 2.98 (s, 1H, -C≡C-H), 2.41 (s, 3H, $-SCOCH_3$), 1.32 (t, *J* = 6.0 Hz, 4H), 1.14 (d, *J* = 1.2 Hz, 18H), 0.66-0.53 (m, 8H) .

^{13}C NMR (100 MHz, Chloroform-*d*) δ 193.8, 158.2, 157.9, 141.7, 141.6, 134.9, 134.1, 132.6, 132.3, 131.8, 130.1, 129.9, 129.3, 129.2, 129.1, 128.4, 127.8, 127.8, 127.6, 125.1, 123.1, 122.3, 120.3, 120.2, 120.0, 119.8, 116.1, 116.1, 112.2, 112.0, 93.9, 92.6, 84.2, 81.8, 78.9, 78.8, 69.5, 31.0, 30.4, 29.7, 29.7, 29.4, 28.7, 27.1, 27.0.

MS (MALDI-TOF, m/z): calcd. for $C_{58}H_{54}O_3S$: 830.379; found 830.382.

UV-vis (CH_3CN): λ_{max} (ε) = 306 (50330), 329 (60432), 344 (76928), 397 (4287), 419 (7715), 444 (12822), 472 nm (15802 M^{-1} cm^{-1}).

Synthesis of *rac*-Ant-C8

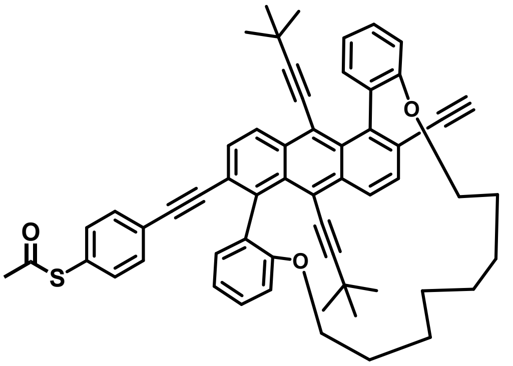

***Rac*-Ant-C8** was synthesized according to the same procedure as was used for ***P*-3-C8**, starting from **rac-1-C8** (40 mg, 0.058 mmol), (4-iodophenyl)ethanethioate (19.6 mg, 0.070 mmol), $Pd(PPh_3)_4$ (3.3 mg, 0.003 mmol) and CuI (1.1 mg, 0.006 mmol). Triethylamine (6 mL). **rac - Ant-C8** was isolated as a deep yellow solid (15 mg, yield = 42.0 %) and recovered **rac -1-C8** (10 mg, 25.3 %).

^{1}H NMR (400 MHz, $CDCl_3$) δ 8.67 (dd, *J* = 8.1, 1.1 Hz, 2H), 7.61 (d, *J* = 8.0, 1H), 7.57 (d, *J* = 8.0, 1H), 7.46 – 7.38 (m, 4H), 7.30 – 7.26 (m, 2H), 7.15 – 7.11 (m, 2H), 7.10-7.05 (m, 2H), 6.96-6.93 (ddd, J = 8.3, 6.0, 1.1 Hz, 2H), 3.97-3.92 (m, 2H), 3.83-3.76 (m, 2H), 2.98 (s, 1H, -C≡C-H), 2.41 (s, 3H, -$SCOCH_3$), 1.32 (t, *J* = 6.0 Hz, 4H), 1.14 (d, *J* = 1.0 Hz, 18H), 0.66-0.53 (m, 8H) .

^{13}C NMR (100 MHz, Chloroform-*d*) δ 193.8, 158.2, 157.9, 141.7, 141.6, 134.9, 134.1, 132.6, 132.3, 132.3, 131.8, 130.1, 129.9, 129.3, 129.2, 129.1, 128.4, 127.8, 127.8, 127.6, 125.1, 123.1, 122.2, 120.3, 120.2, 120.0, 119.9, 116.2, 116.1, 112.2, 112.0, 93.9, 92.6, 84.2, 81.8, 78.9, 78.9, 69.5, 31.0, 30.9, 29.7, 29.7, 29.4, 28.7, 27.1, 27.0.

MS (MALDI-TOF, m/z): calcd. for $C_{58}H_{54}O_3S$: 830.379; found 830.449.

Synthesis of *P*-Ant-C4

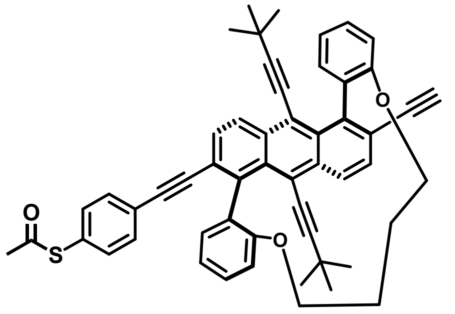

***P*-Ant-C4** was synthesized according to the same procedure as was used for ***P*-Ant-C8**, starting from ***P*-1-C4** (44 mg, 0.070 mmol), (4-iodophenyl)ethanethioate (23.3 mg, 0.084 mmol), $Pd(PPh_3)_4$ (4.6 mg, 0.004 mmol) and CuI (1.3 mg, 0.007 mmol). Triethylamine (6 mL). ***P*-Ant-C4** was isolated as a deep yellow solid (16 mg, yield = 36.2 %) and recovered ***P*-1-C4** (8 mg, 18.3%).

^{1}H NMR (400 MHz, $CDCl_3$) δ 8.34 (dd, J = 9.0, 1.4 Hz, 2H), 7.99 (dd, J = 8.0, 1.6 Hz, 1H), 7.96 (d, J = 8.0, 1H), 7.56 (dd, J = 16.3, 9.0 Hz, 2H), 7.39 – 7.36 (m, 2H), 7.35-7.32 (m, 4H), 7.18 – 7.09 (m, 2H), 6.70 – 6.67 (m, 2H), 3.67-3.65 (m, 2H), 3.34 – 3.29 (m, 2H), 3.11 (s, 1H, -C≡C-H), 2.42 (s, 3H, -$SCOCH_3$), 1.11 (d, J = 1.7 Hz, 18H), 0.90 – 0.77 (m, 2H), 0.57-0.54 (m, 2H).

^{13}C NMR (100 MHz, Chloroform-*d*) δ 193.6, 156.0, 155.9, 140.0, 139.6, 134.1, 133.0, 132.2, 132.1, 132.0, 129.6, 129.4, 129.3, 129.1, 129.1, 128.7, 127.6, 126.2, 126.1, 125.1, 121.5, 120.6, 119.4, 119.2, 118.6, 113.9, 110.1, 110.0, 93.2, 92.7, 84.4, 81.8, 66.6, 30.9, 30.4, 29.8, 28.5, 26.1.

MS (MALDI-TOF, m/z): calcd. for $C_{54}H_{46}O_3S$: 774.316; found 774.367.

UV-vis (CH_3CN): λ_{max} (ε) = 313 (49720), 332 (51831), 346 (64459), 402 (4350), 425 (7567), 450 (11893), 480 nm (14309 M^{-1} cm^{-1}).

Synthesis of ***M*-Ant-C4**

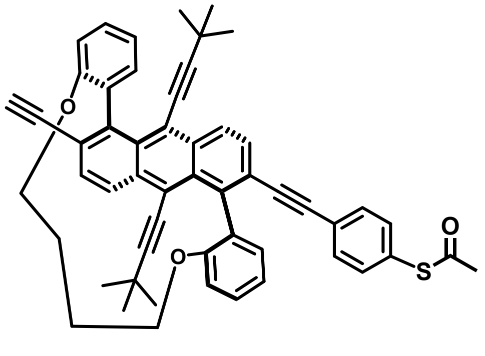

***M*-Ant-C4** was synthesized according to the same procedure as was used for ***P*-Ant-C8**, starting from ***M*-1-C4** (44 mg, 0.070 mmol), (4-iodophenyl)ethanethioate (23.3 mg, 0.084 mmol), $Pd(PPh_3)_4$ (4.6 mg, 0.004 mmol) and CuI (1.3 mg, 0.007 mmol). Triethylamine (6 mL). ***M*-Ant-C4** was isolated as a deep yellow solid (17 mg, yield = 39.9 %) and recovered ***M*-1-C4** (9 mg, 24.6%).

^{1}H NMR (400 MHz, $CDCl_3$) δ 8.34 (dd, J = 9.0, 1.4 Hz, 2H), 8.02 (dd, J = 8.0, 1.6 Hz, 1H), 7.96 (dd, J = 8.0, 1H), 7.58 (d, J = 8.0, 1H), 7.54 (d, J = 8.0, 1H), 7.39 – 7.35 (m, 2H), 7.34-7.32 (m, 4H), 7.19 – 7.09 (m, 2H), 6.70 – 6.67 (m, 2H), 3.67-3.65 (m, 2H), 3.34 – 3.29 (m, 2H), 3.11 (s, 1H, -C≡C-H), 2.42 (s, 3H, $-SCOCH_3$), 1.11 (d, J = 1.7 Hz, 18H), 0.88 – 0.77 (m, 2H), 0.57-0.54 (m, 2H).

^{13}C NMR (100 MHz, Chloroform-*d*) δ 193.6, 156.1, 156.0, 140.0, 139.7, 134.2, 133.2, 132.4, 132.2, 132.1, 132.0, 129.7, 129.5, 129.4, 129.3, 129.3, 128.9, 127.8, 126.3, 126.3, 125.2, 121.6, 120.8, 119.5, 119.4, 118.8, 114.0, 110.3, 110.2, 93.4, 92.8, 84.3, 81.7, 65.9, 30.8, 30.3, 29.7, 28.4, 25.9.
MS (MALDI-TOF, m/z): calcd. for $C_{54}H_{46}O_3S$: 774.316; found 774.341.

UV-vis (CH_3CN): λ_{max} (ε) = 313 (49720), 332 (51831), 346 (64459), 402 (4350), 425 (7567), 450 (11893), 480 nm (14309 M^{-1} cm^{-1}).

Synthesis of *rac*-Ant-C4

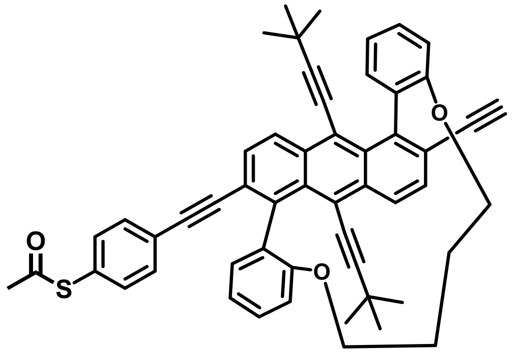

***Rac*-Ant-C4** was synthesized according to the same procedure as was used for ***P*-Ant-C8**, starting from ***rac*-1-C4** (40 mg, 0.064 mmol), (4-iodophenyl)ethanethioate (21 mg, 0.076 mmol), $Pd(PPh_3)_4$ (3.6 mg, 0.003 mmol) and CuI (1.2 mg, 0.006 mmol). Triethylamine (6 mL). ***rac*-Ant-C4** was isolated as a deep yellow solid (14 mg, yield = 34.6 %) and recovered **rac -1-C4** (8 mg, 24.1 %).

^{1}H NMR (400 MHz, $CDCl_3$) δ 8.34 (dd, J = 9.0, 1.4 Hz, 2H), 8.02 (dd, J = 7.5, 1.7 Hz, 1H), 7.96 (dd, J = 7.5, 1H), 7.56 (dd, J = 16.4, 9.0 Hz, 2H), 7.39 – 7.36 (m, 2H), 7.34-7.32 (m, 4H), 7.19 – 7.09 (m, 2H), 6.70 – 6.67 (m, 2H), 3.67-3.65 (m, 2H), 3.34 – 3.29 (m, 2H), 3.11 (s, 1H, -C≡C-H), 2.42 (s, 3H, $-SCOCH_3$), 1.11 (d, J = 1.7 Hz, 18H), 0.88 – 0.77 (m, 2H), 0.57-0.54 (m, 2H).

^{13}C NMR (100 MHz, Chloroform-*d*) δ 193.6, 156.1, 156.0, 140.0, 139.7, 135.3, 133.2, 132.4, 132.2, 132.1, 132.0, 129.7, 129.5, 129.4, 129.3, 129.3, 128.9, 127.8, 126.3, 126.3, 125.2, 121.6, 120.8, 119.5, 119.4, 118.8, 114.0, 110.3, 110.2, 93.4, 92.8, 84.3, 81.9, 66.0, 30.9, 30.4, 28.5, 28.4, 26.1.
MS (MALDI-TOF, m/z): calcd. for $C_{54}H_{46}O_3S$: 774.316; found 774.383.

S3 Characterization

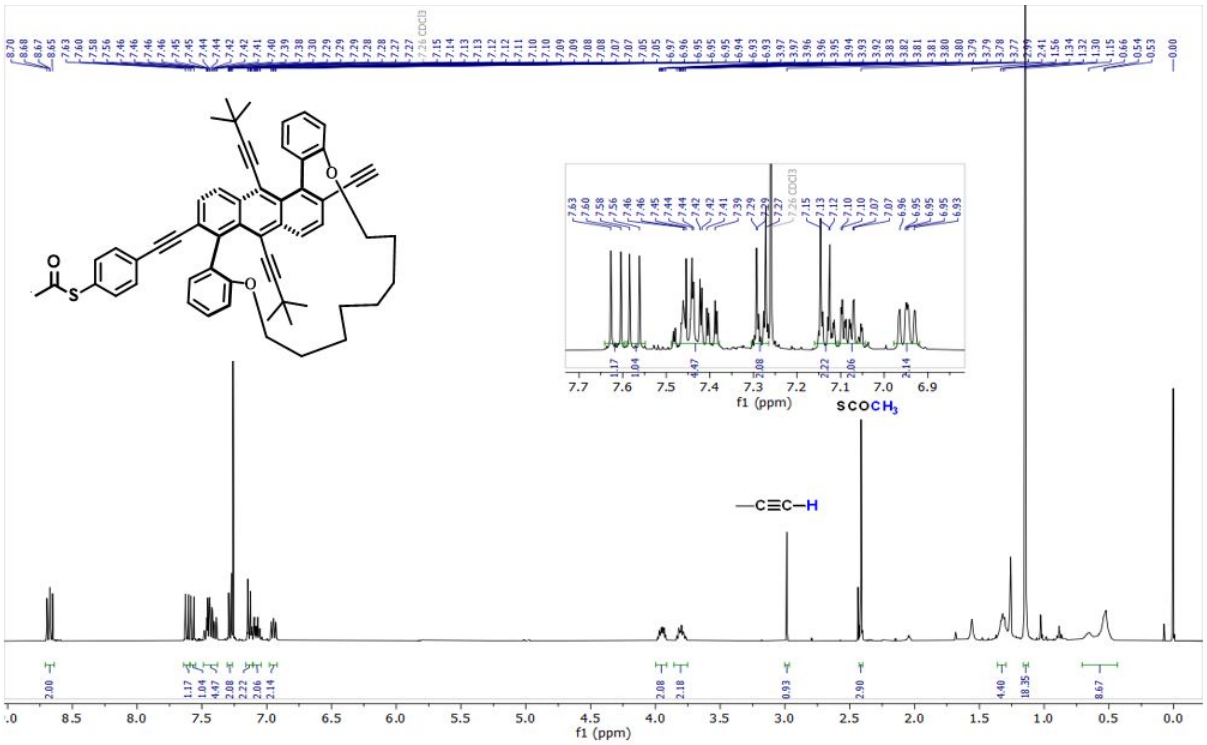

Figure S1. ^{1}H NMR (400 MHz) of ***P*-Ant-C8** in $CDCl_3$, measured at 298 K.

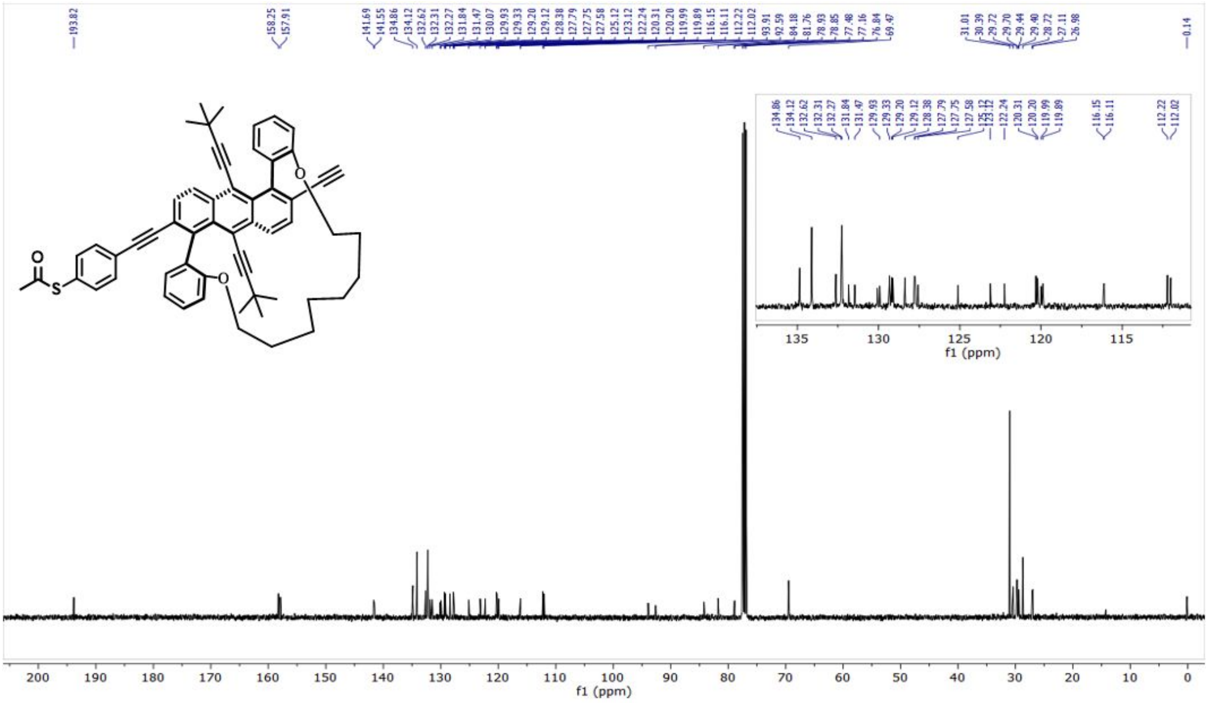

Figure S2. ^{13}C NMR (100 MHz) of ***P*-Ant-C8** in $CDCl_3$, measured at 298 K.

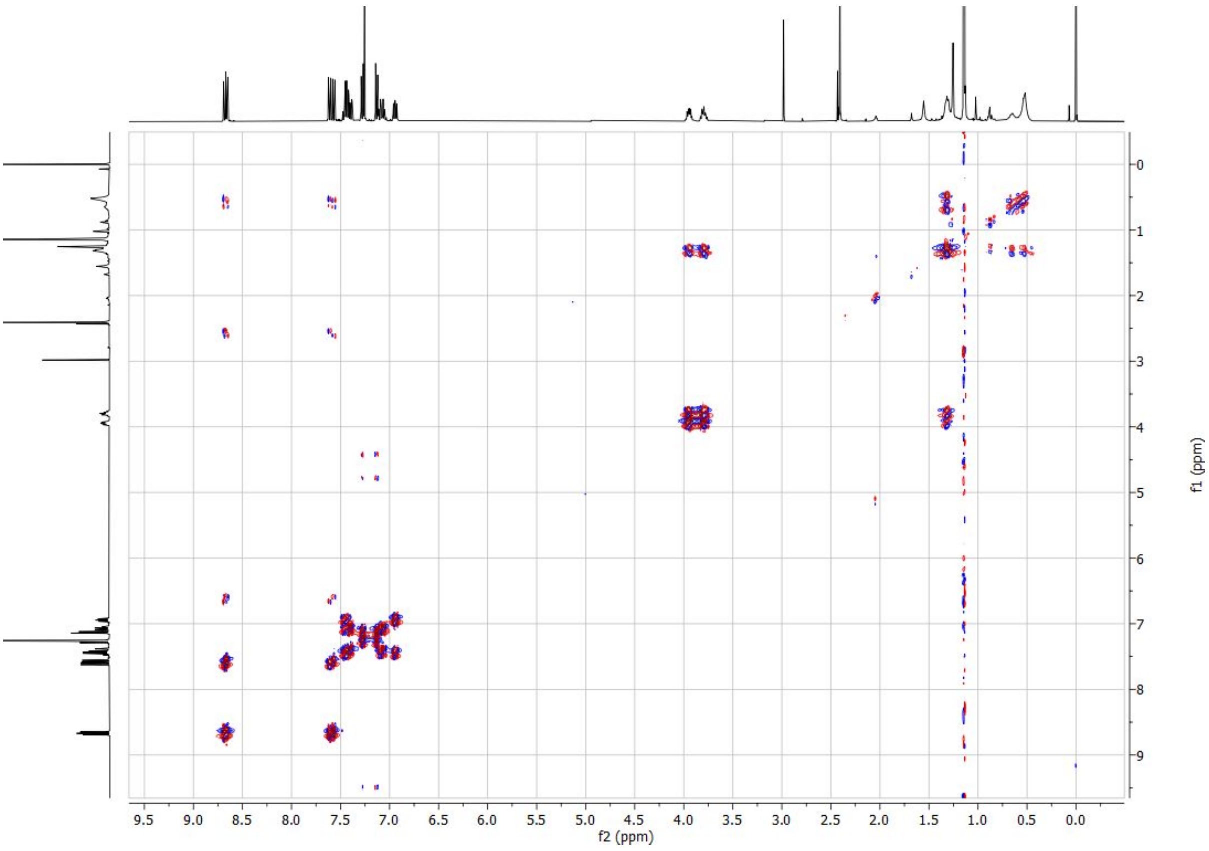

Figure S3. COSY NMR (400 MHz) of ***P*-Ant-C8** in $CDCl_3$, measured at 298 K.

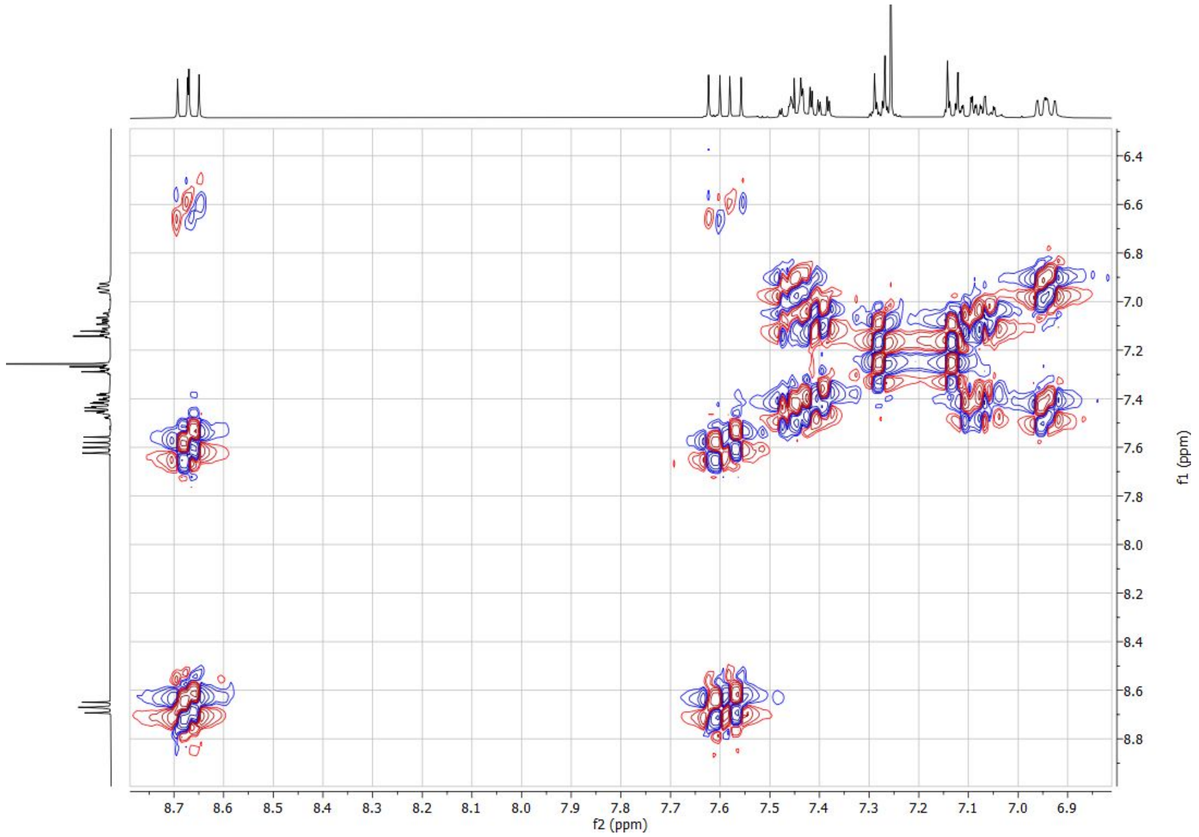

Figure S4. COSY NMR (400 MHz) of ***P*-Ant-C8** in $CDCl_3$, measured at 298 K (expansion in aromatic region).

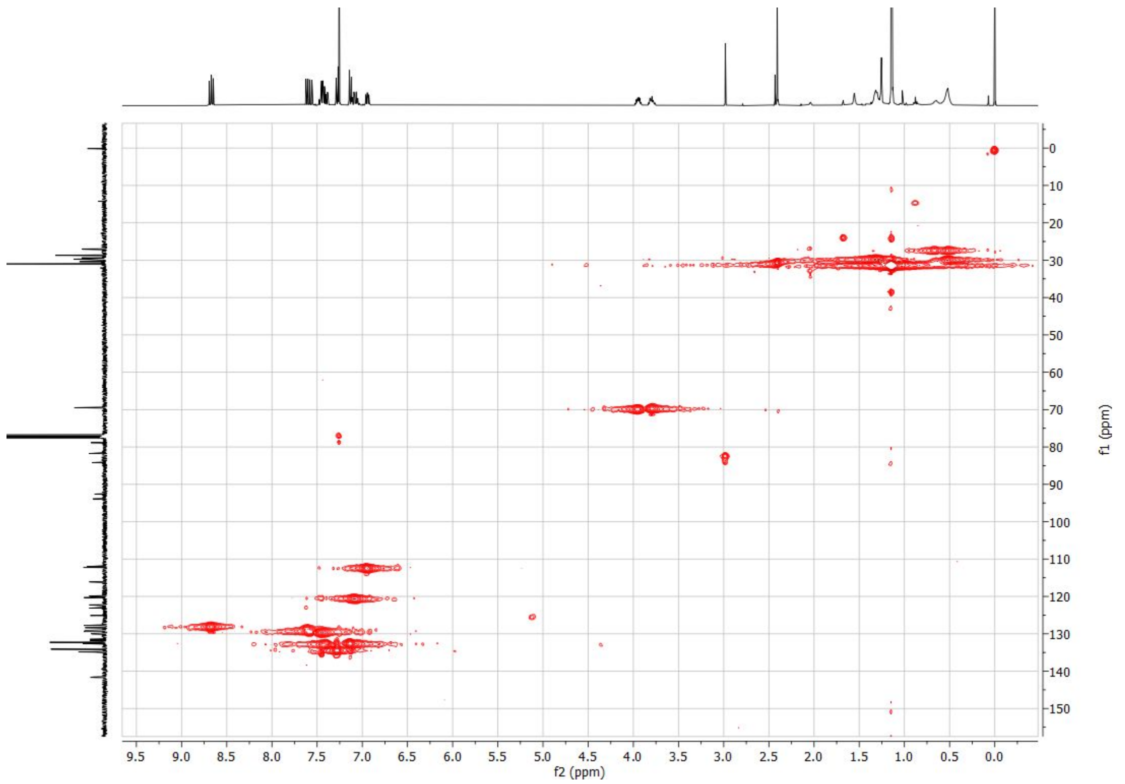

Figure S5. HSQC NMR (400 MHz) of ***P*-Ant-C8** in $CDCl_3$, measured at 298 K.

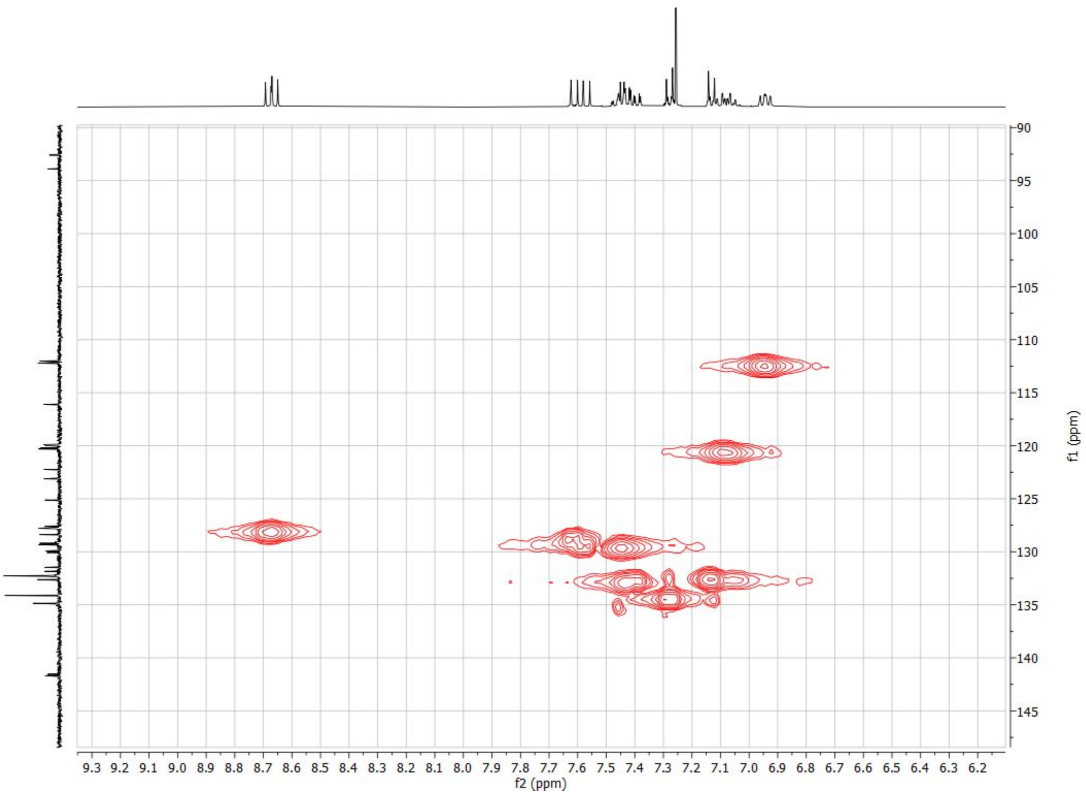

Figure S6. HSQC NMR (400 MHz) of ***P*-Ant-C8** in $CDCl_3$, measured at 298 K (expansion in aromatic region).

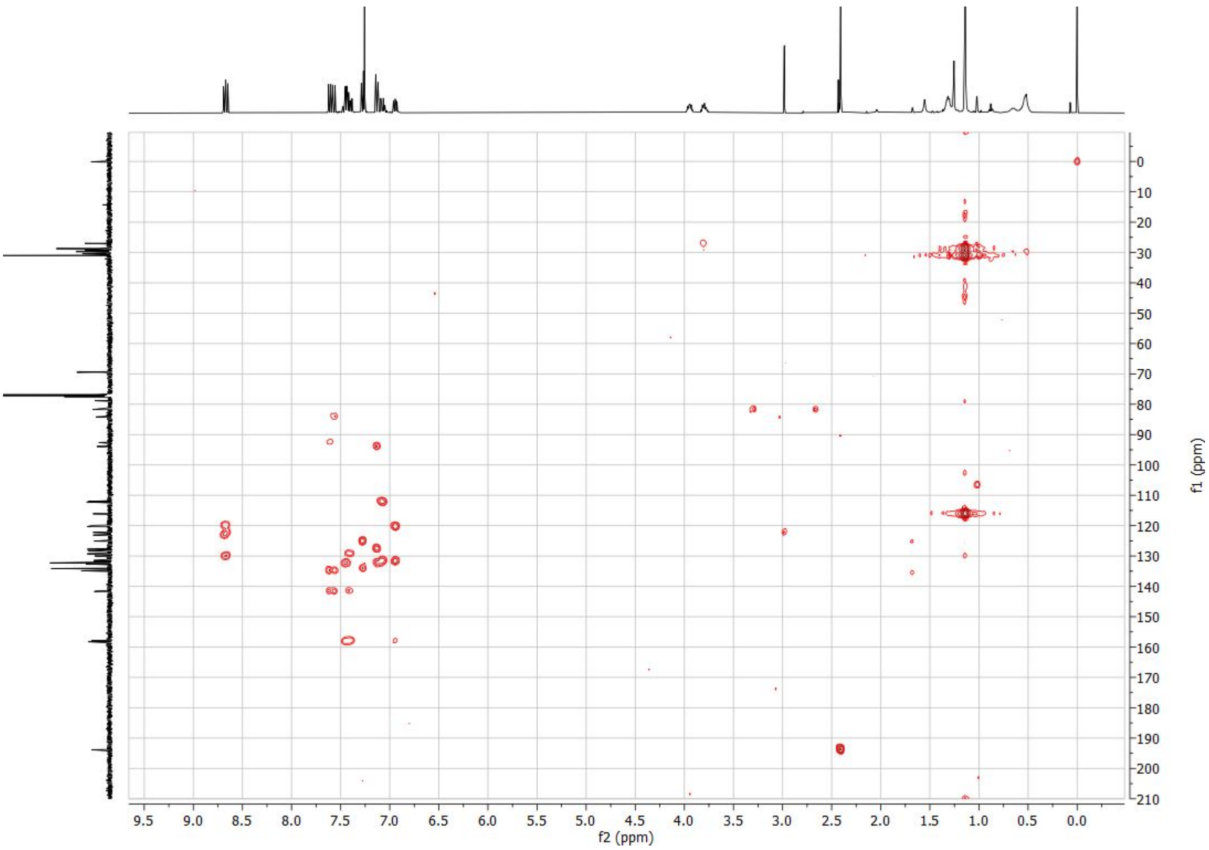

Figure S7. HMBC NMR (400 MHz) of ***P*-Ant-C8** in $CDCl_3$, measured at 298 K.

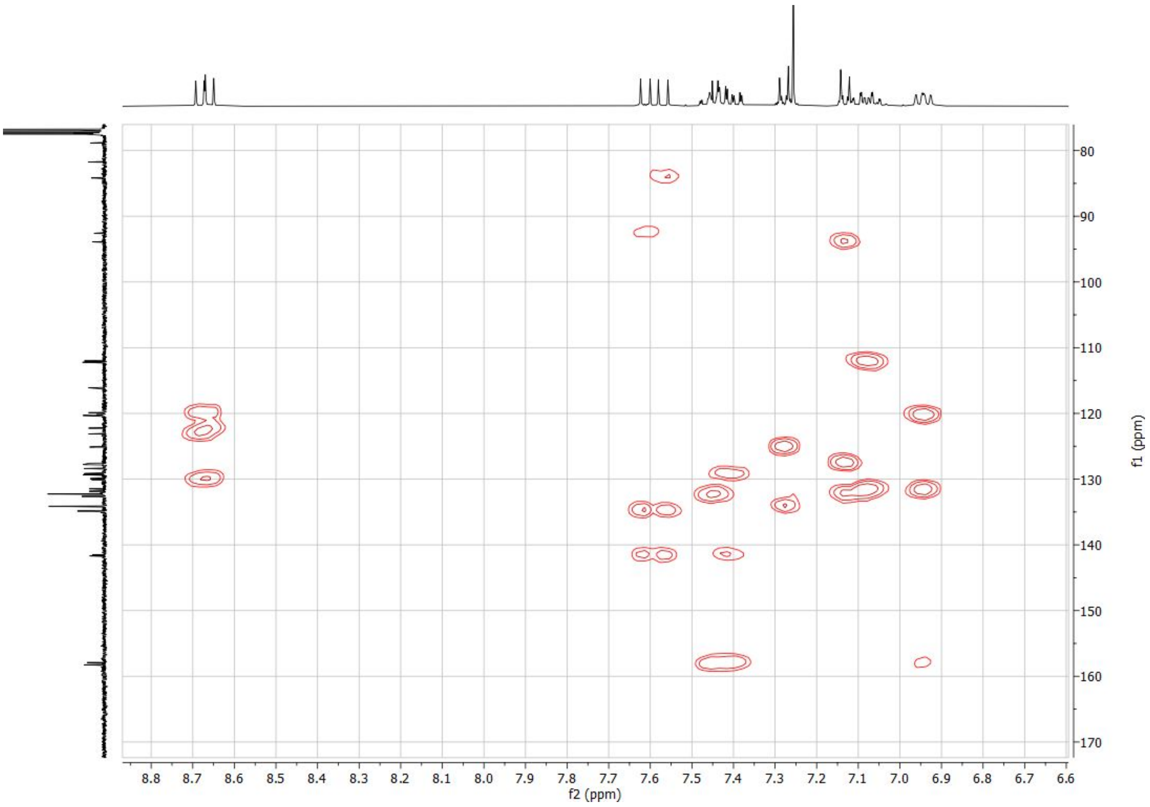

Figure S8. HMBC NMR (500 MHz) of ***P*-Ant-C8** in $CDCl_3$, measured at 298 K (expansion in aromatic region).

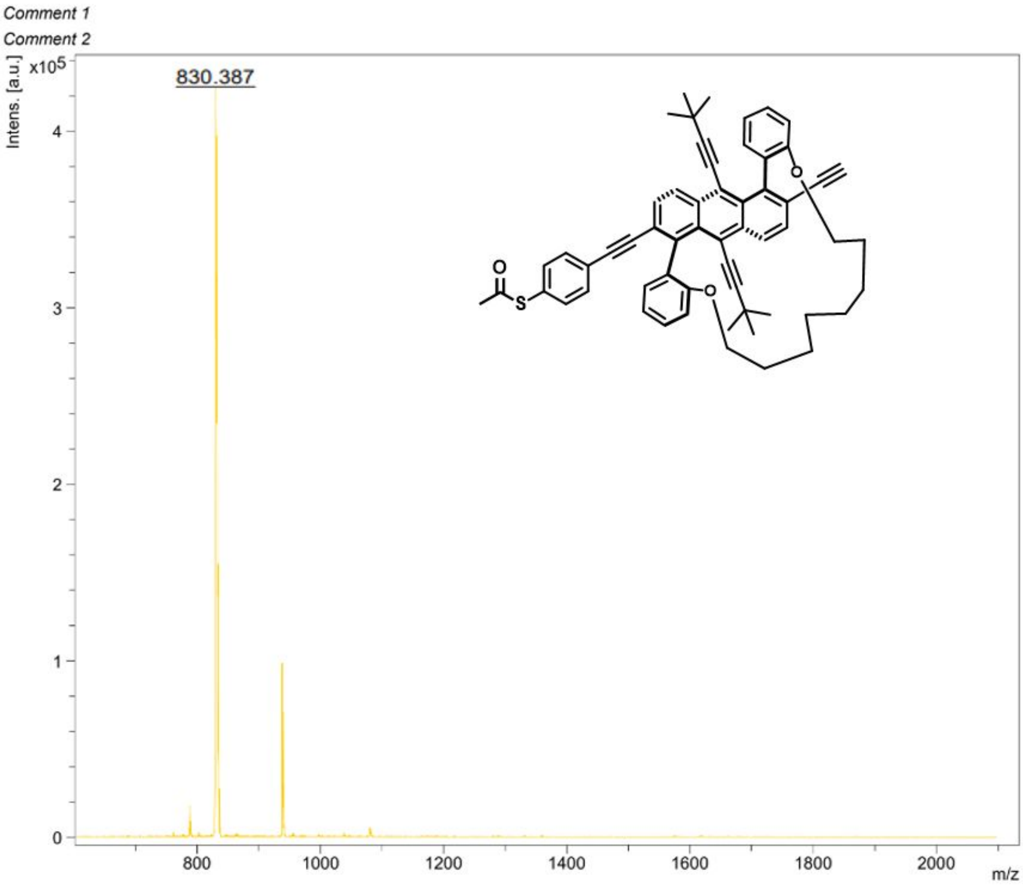

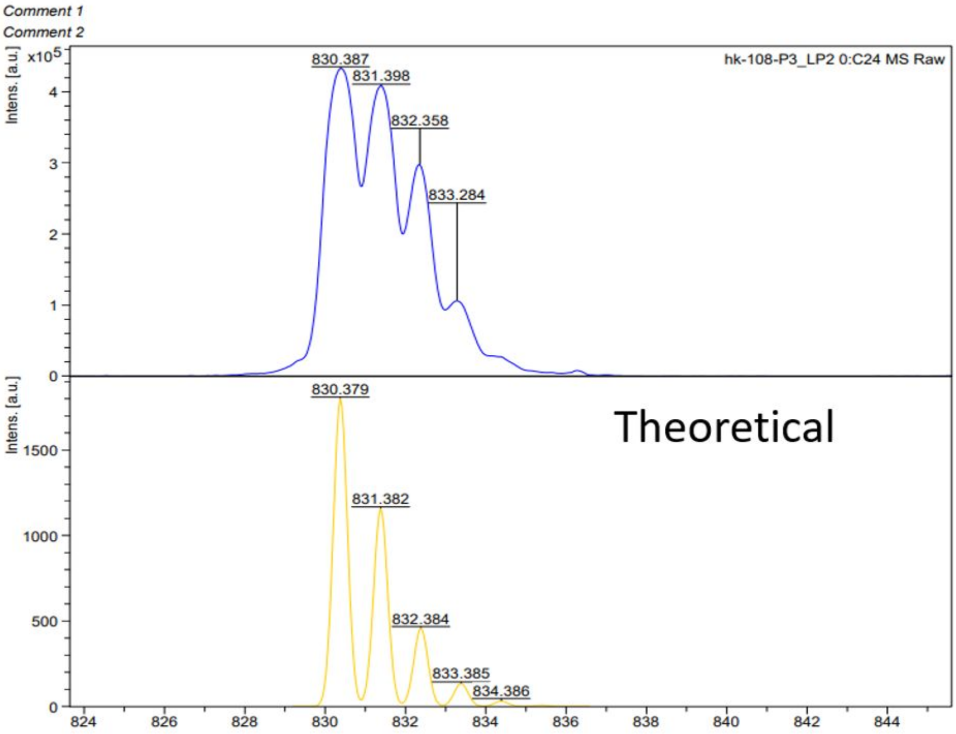

Figure S9. MALDI-TOF of ***P*-Ant-C8** in matrix DCTB

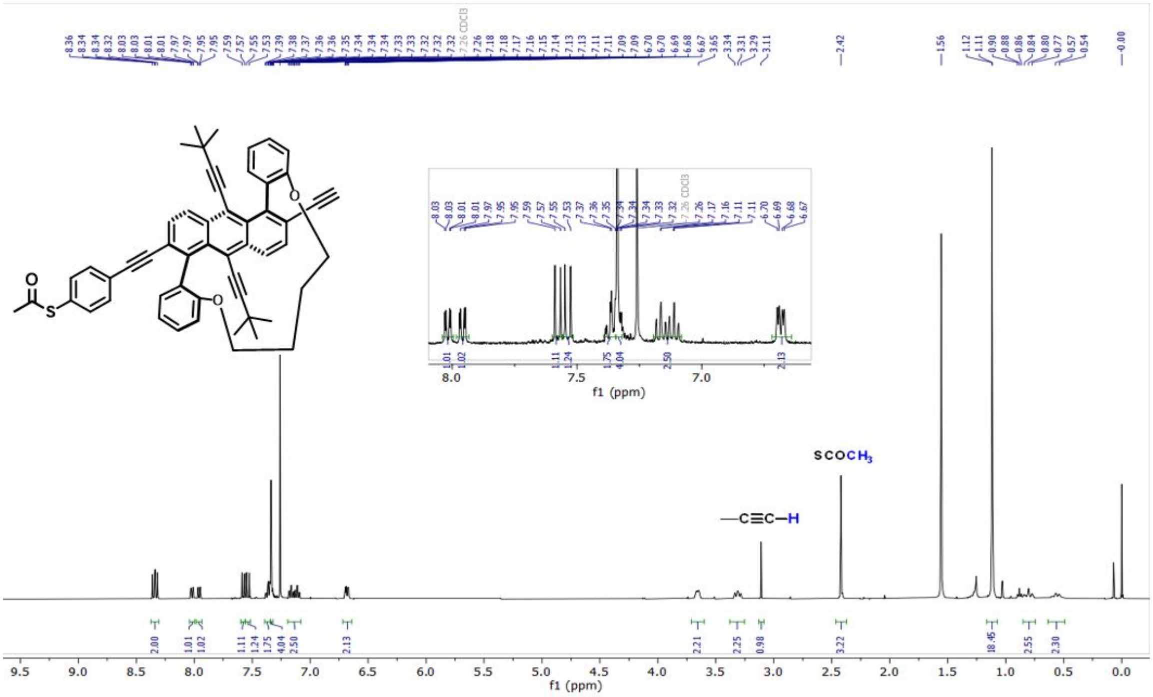

Figure S10. ^{1}H NMR (400 MHz) of ***P*-Ant-C4** in $CDCl_3$, measured at 298 K.

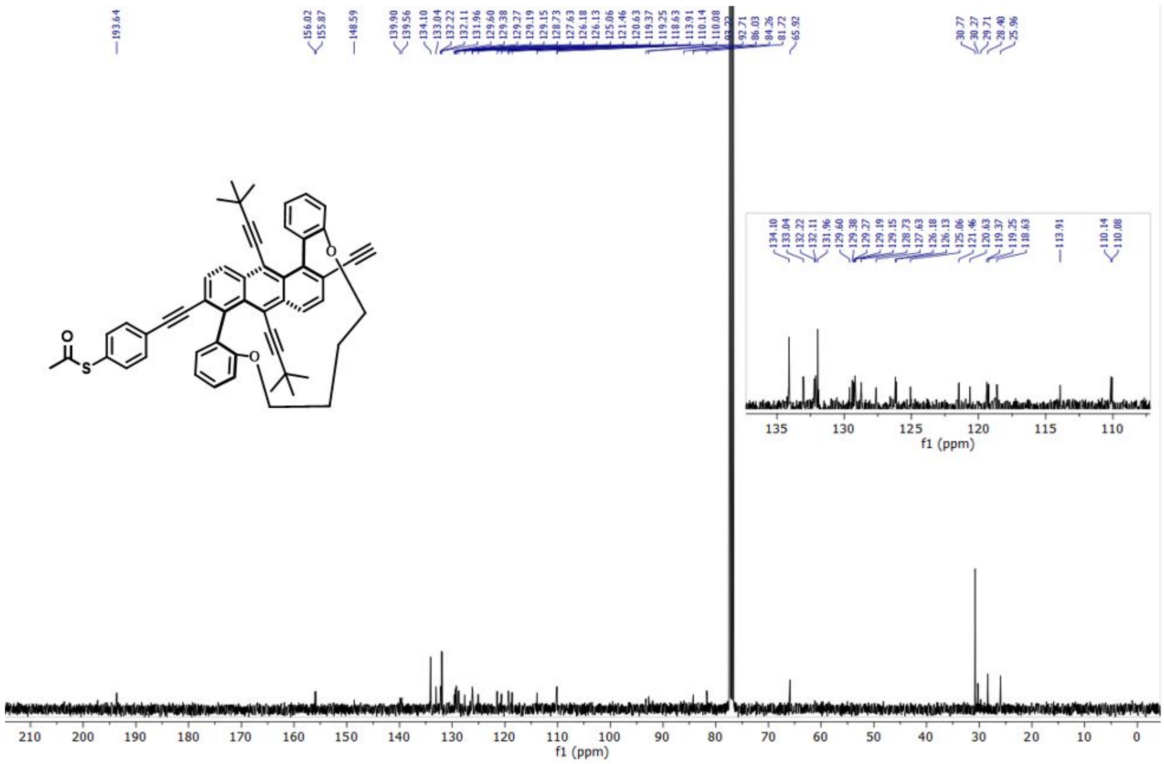

Figure S11. ^{13}C NMR (100 MHz) of ***P*-Ant-C4** in $CDCl_3$, measured at 298 K.

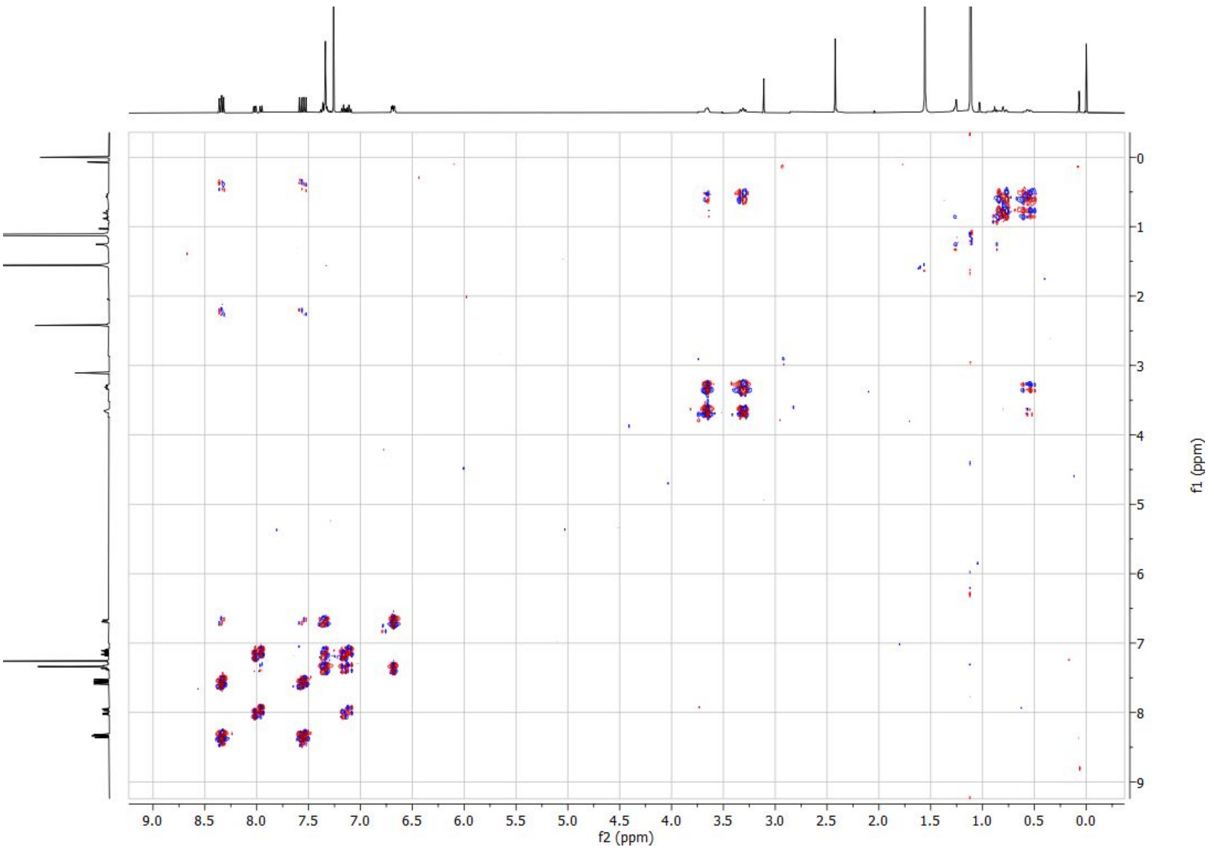

Figure S12. COSY NMR (400 MHz) of ***P*-Ant-C4** in $CDCl_3$, measured at 298 K.

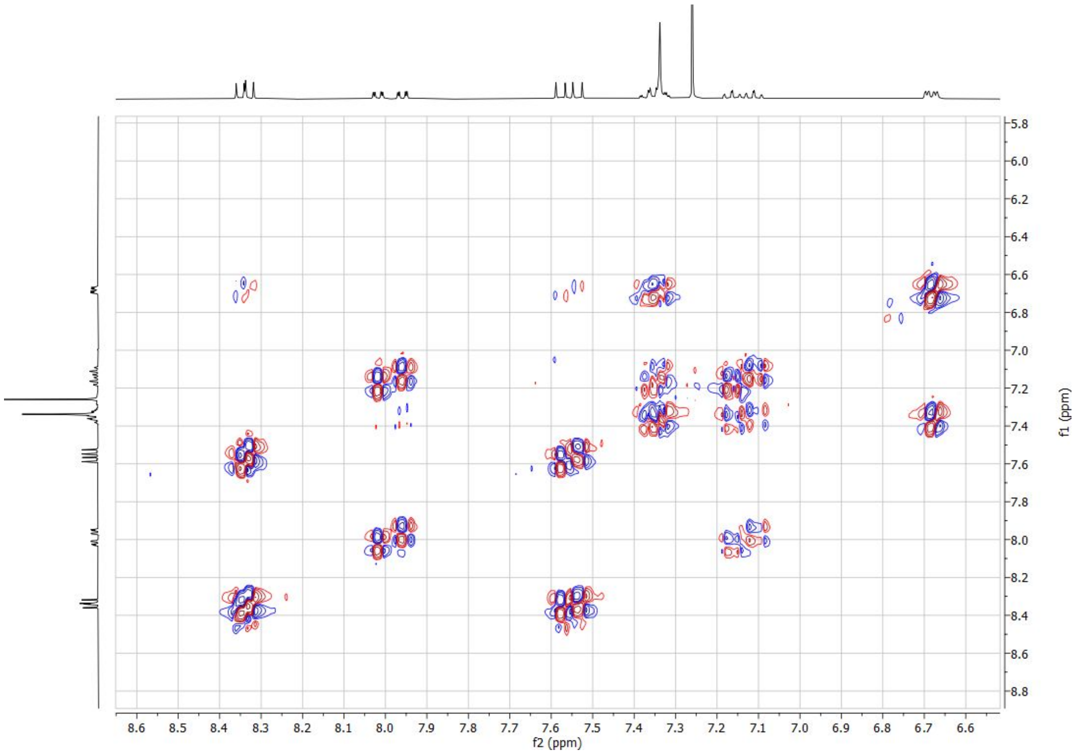

Figure S13. COSY NMR (400 MHz) of ***P*-Ant-C4** in $CDCl_3$, measured at 298 K (expansion in aromatic region).

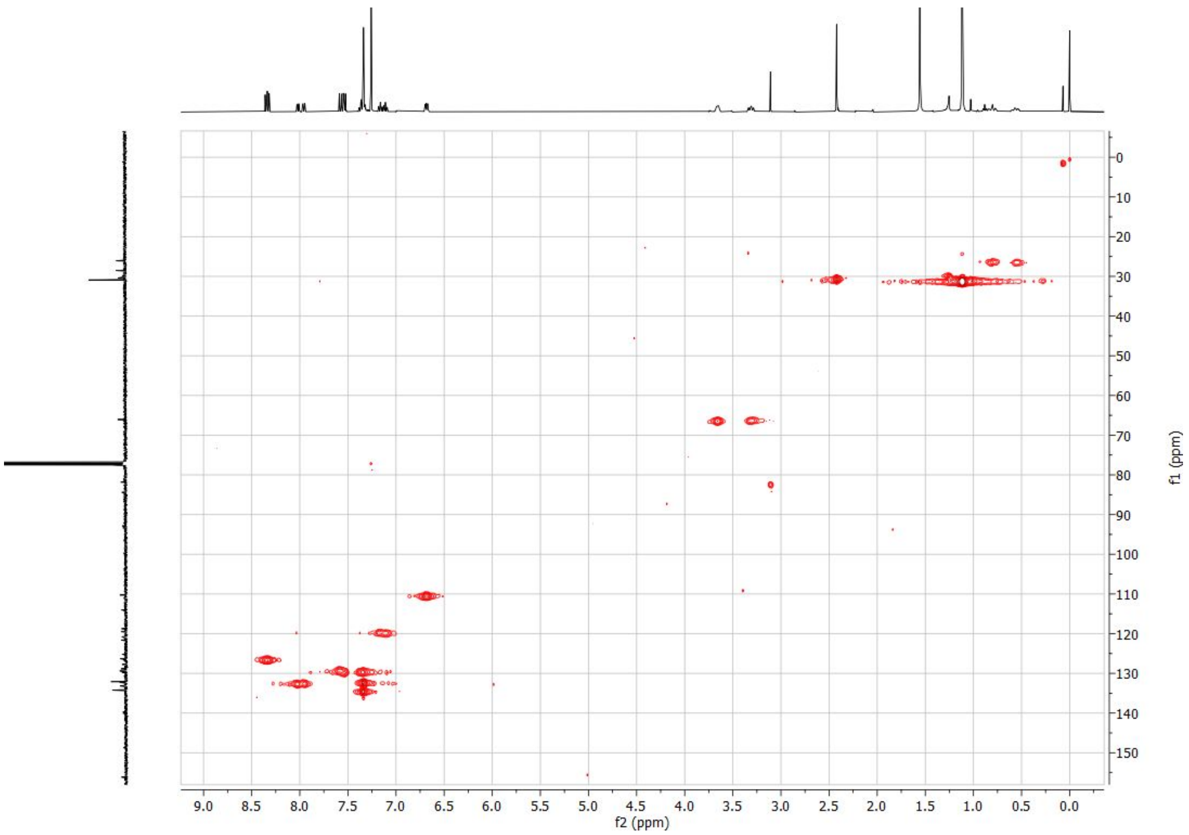

Figure S14. HSQC NMR (400 MHz) of ***P*-Ant-C4** in $CDCl_3$, measured at 298 K.

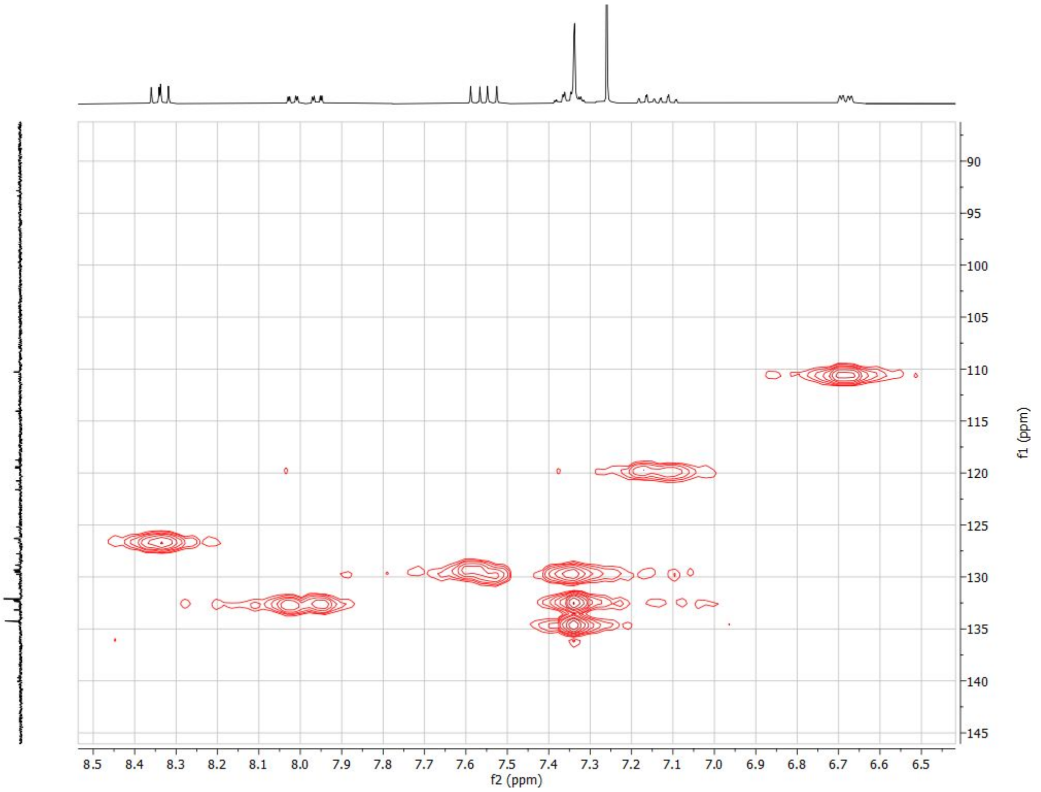

Figure S15. HSQC NMR (400 MHz) of ***P*-Ant-C4** in $CDCl_3$, measured at 298 K (expansion in aromatic region).

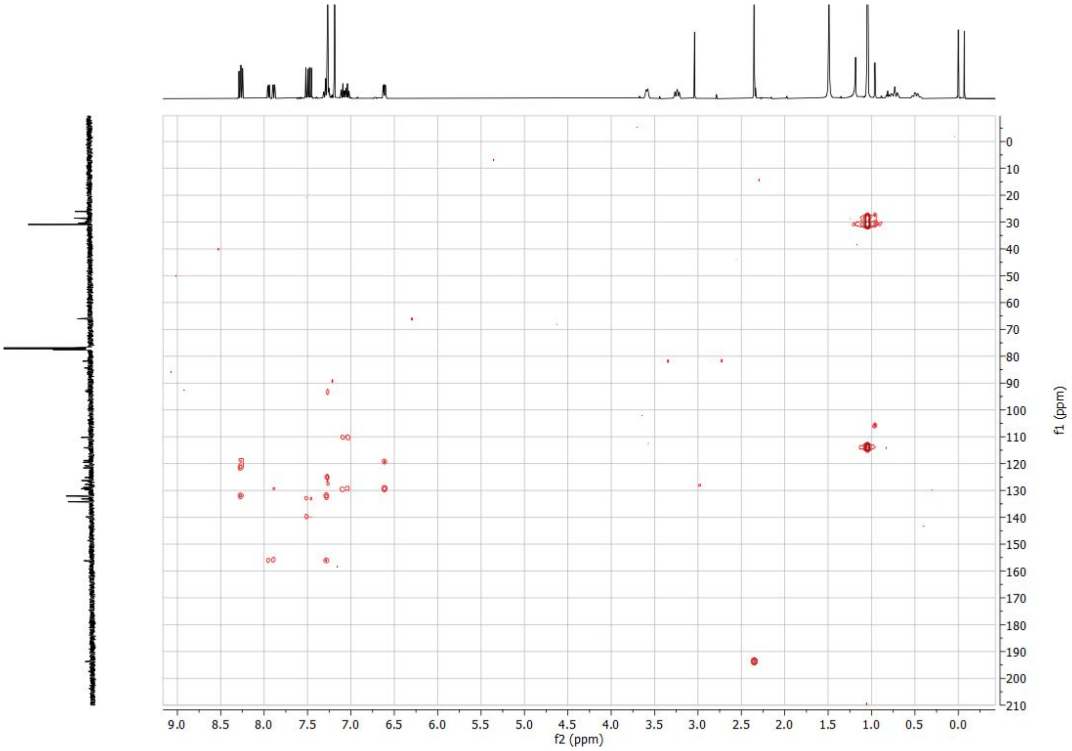

Figure S16. HMBC NMR (400 MHz) of ***P*-Ant-C4** in $CDCl_3$, measured at 298 K.

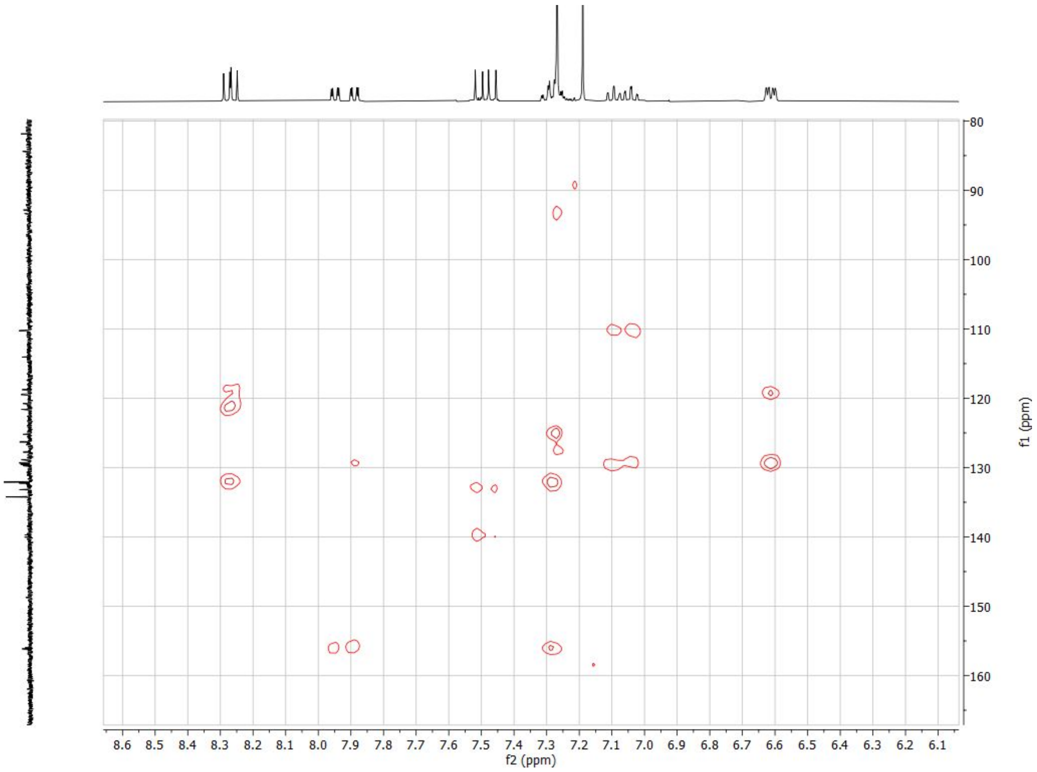

Figure S17. HMBC NMR (400 MHz) of ***P*-Ant-C4** in $CDCl_3$, measured at 298 K (expansion in aromatic region).

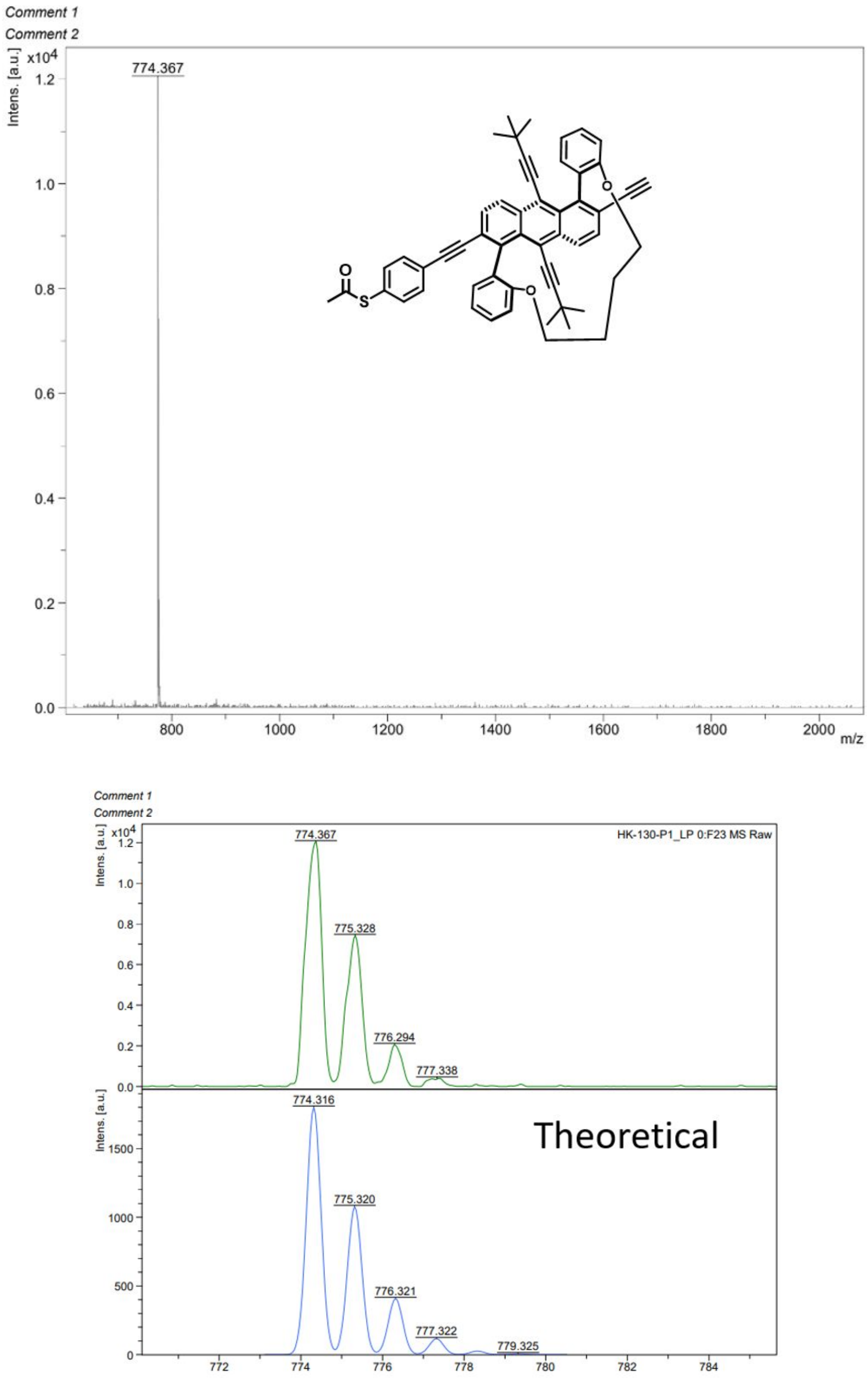

Figure S18. MALDI-TOF of ***P*-Ant-C4** in DCTB matrix.

S4 Photophysical properties

All photophysical studies were performed with dilute solutions of the compounds keeping the absorbance from the lowest energy band in the range of 0.05 to exclude self-absorption

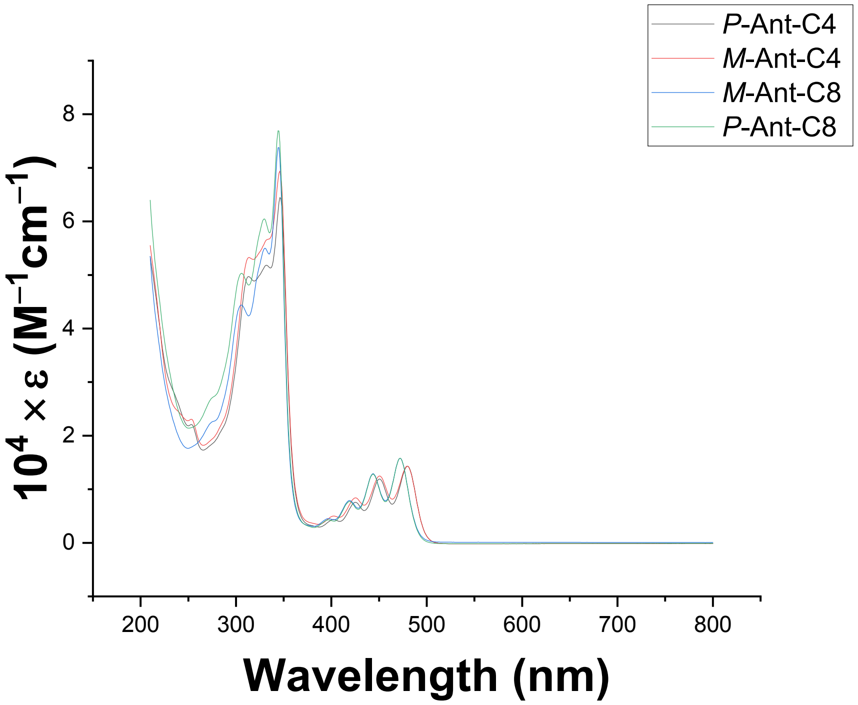

Figure S19. UV-vis absorption spectra of compounds ***P*-Ant-C4**, ***M*-Ant-C4**, ***M*-Ant-C8**, ***P*-Ant-C8** in acetonitrile at 298 K.

	Atomic conc. [%]	Error [%]	Mass conc. [%]	Error [%]
**Au 4f**	38,29	0,70	90,35	0,26
**S 2p**	0,71	0,21	0,27	0,08
**C 1s**	48,45	0,94	6,97	0,24
**O 1s**	12,54	0,49	2,40	0,09

Table S20: Summary of XPS results collected from Au surface after thioester attachment, demonstrating an organic layer of 2 nm thick, with a clear sulfur signal.

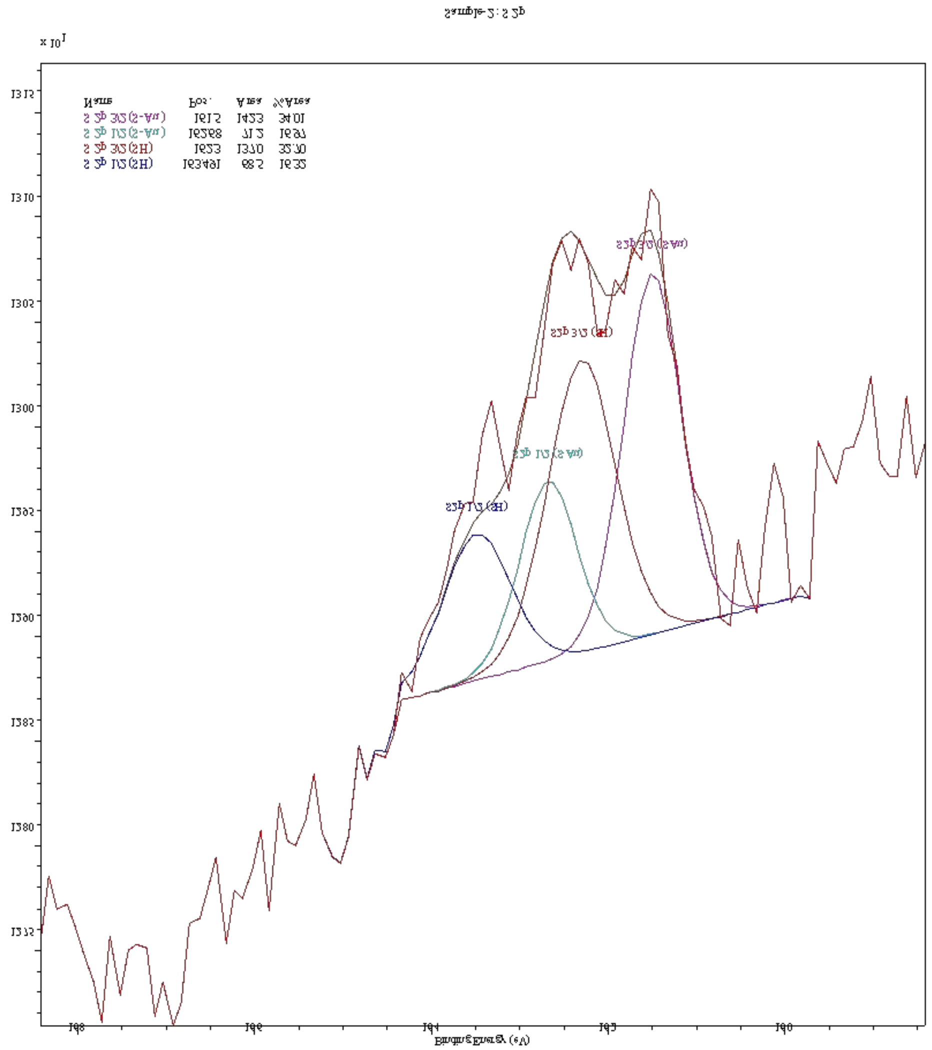

Figure S21: XPS spectra collected from Au surface after thioester attachment. In this case there are two thioesters on each molecule, therefore two types of thioesters are shown: one on gold and the other is free.

S5 Preparation of self-assembled monolayers for CD measurements:

The substrate for CD measurements were prepared using e-beam evaporation deposition technique. An 8 nm on Ti layer and 10 nm of gold layer were deposited on quartz substrates. The substrates were cleaned by immersing them first in boiling acetone and then in ethanol for 10 minutes, each. The self-assembled monolayers were prepared on these surfaces by immersing the surfaces into the respective compound solutions of 0.2mg/ml for

48 hours. Three substrates were combined together for CD measurements for each case. Also M and P-C8-Thio-Ac compounds were drop casted on quartz substrates for CD measurements.

S6 Atomic force microscopy with magnetic conducting probe (mc-AFM):

S6.1 Sample preparation for magnetic- conductive atomic force microscopy (mc-AFM):

The substrates for the mc-AFM studies were prepared using the e-beam evaporation deposition technique. A 100 nm layer of Ni layer is sputtered, followed by a 8 nm layer of Au layer on a Si wafer, with a 10 nm Ti layer as an adhesion layer. The deposited multilayer surfaces were cleaned by immersing them first in boiling acetone and then in ethanol for 10 minutes. The surfaces were also cleaned with UV ozone for 15 minutes, followed by a final 45-minute incubation in warm ethanol. The role of the Ni/Au surfaces is to allow spin polarization of electrons injected from the surface into the chiral molecules induced by a magnetic field. The self-assembled monolayers were deposited on the Ni/Au surfaces by incubating the surfaces into the respective solutions of 0.2mg/ml for 48 hours.

S6.2 Measurement using mc-AFM:

Magnetic field-dependent current-voltage (*I-V*) characteristics of the prepared samples were determined using a multimodal scanning magnetic probe microscopy (SPM) system equipped with a Beetle Ambient AFM and an electromagnet with R9 electronic controller (RHK Technology). Voltage spectroscopy for the *I-V* measurements was performed by applying voltage ramps with a non-magnetic Pt tip (DPE-XSC11, μmasch with spring constant 3-5 Nm^{-1}) in contact mode. At least 50 *I-V* curves were scanned in an applied magnetic field of 0.50 T for both magnetic field orientations (field UP and DOWN) and representative plots for (*P*) and (*M*) enantiomers on Ni/Au and ITO surfaces are depicted in Figure S1 and S2, respectively.

S6.3 Spin dependent transport properties for (M) and (P) enantiomers on Ni/Au surfaces:

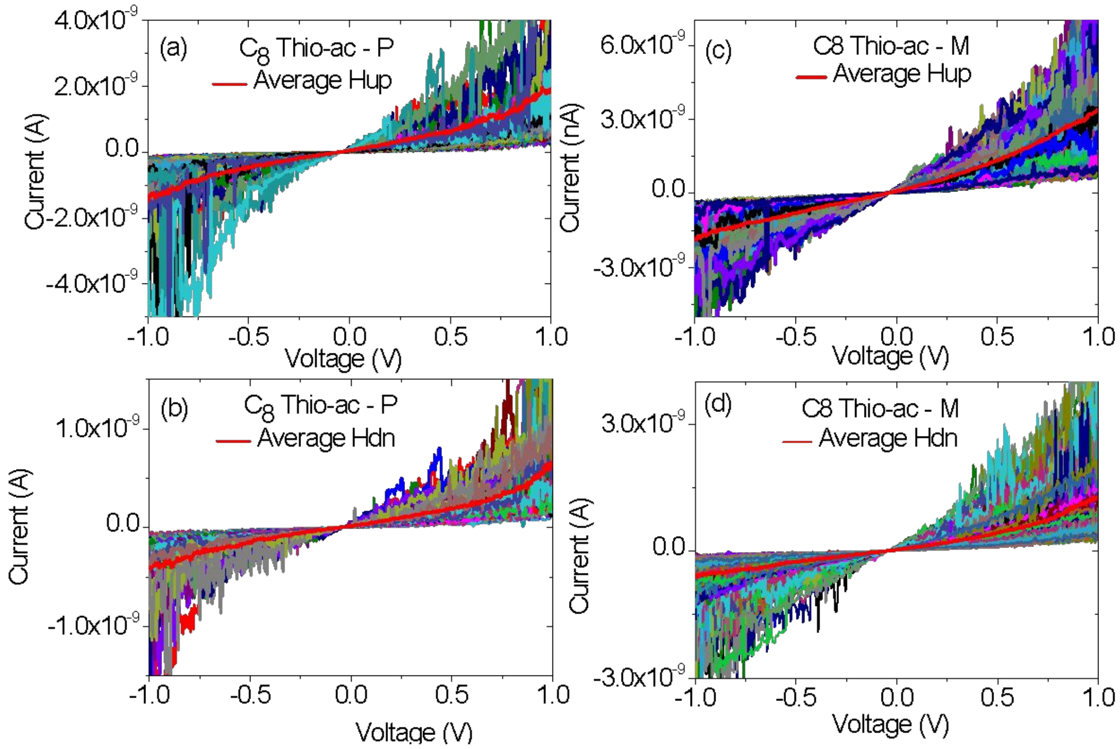

Figure S22: Spin dependent transport properties for (*M*) and (*P*) enantiomers on the Ni/Au surfaces with the magnet north pole pointing up (a&c) or down (b&d) respectively. At least 50 *I-V* curves were scanned in an applied magnetic field of 0.50 T for both magnetic field orientation (field UP and DOWN). The bold red curve represents the average over more than 50 *I-V* curves.

S6.4 Spin dependent transport properties for (M) and (P) enantiomers on ITO surfaces:

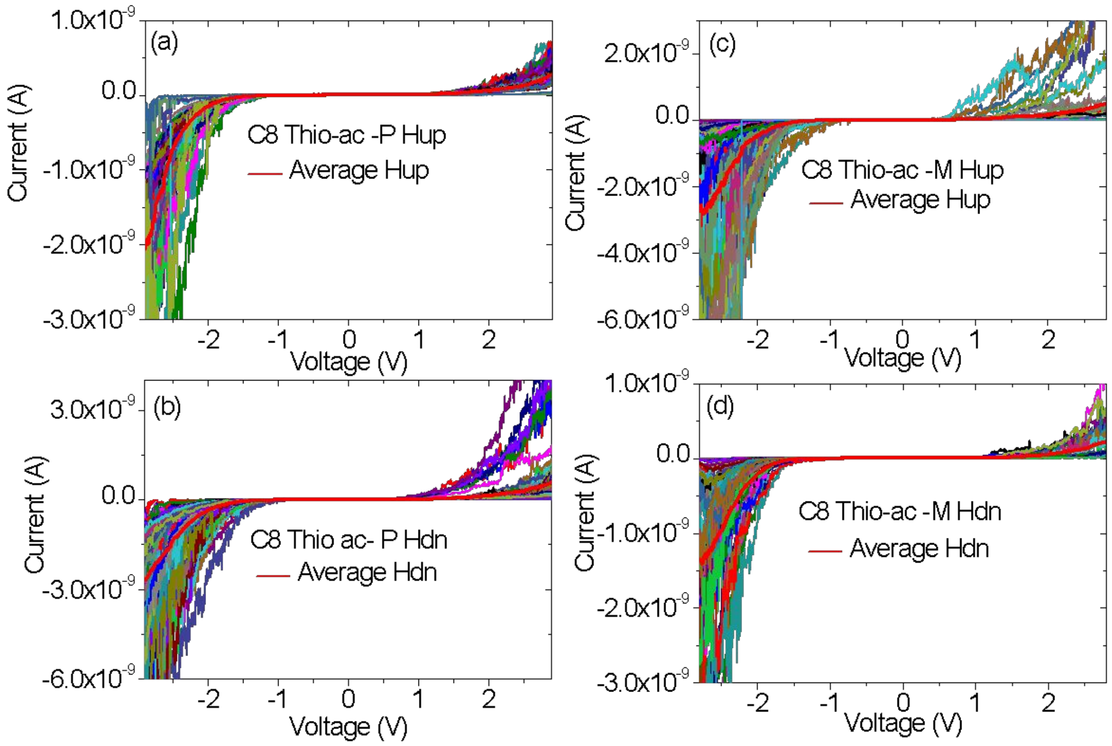

Figure S23: Spin dependent transport for (*M*) and (*P*) enantiomers on ITO surfaces with the magnet north pole pointing up (a&c) or down (b&d) respectively. The bold red curve represents the average over more than 50 *I-V* curves.

S7 Methodology

S7.1 Geometry optimization

Optimized geometries for both enantiomers were obtained using density functional theory (DFT) with the CAM-B3LYP functional and Dunning's correlation consistent basis set cc-pVDZ. The Resolution-of-Identity (RI) scheme for Coulomb term and seminumerical integration for exchange term (RIJCOSX) was used to reduce the computational costs of the geometry optimization.

S7.2 Excited state calculations

Excitation energies, with corresponding oscillator and rotatory strengths, were computed at the TD-CAM-B3LYP/cc-pVDZ level of theory. The RIJCOSX approximation was used to reduce the computational cost. In order to simulate the transition magnetic moment, the gauge origin was placed in the center of mass of the molecule.

Table S21: Transition Dipole Magnetic Moments of first 5 excited states for (M)-enantiomer.

1	0.12346<br>(-0.12344)	-0.18162<br>(-0.18166)	0.36471<br>(0.36464)
2	0.17278<br>(-0.17279)	-0.12132<br>(0.12138)	-0.00098<br>(0.00093)
3	-0.24074<br>(0.24082)	0.20457<br>(-0.20472)	-0.27472<br>(0.27556)
4	-0.06144<br>(0.06151)	0.09980<br>(-0.09998)	-0.09311<br>(0.09341)
5	-0.58102<br>(-0.58123)	0.53751<br>(0.53769)	0.07641<br>(0.07682)

Table S22: Transition Dipole Electric Moments and oscillator strength (f) of first 5 excited states for (M)-enantiomer.

State	X component	Y component	Z component	f
1	1.31457	-1.65865	-0.02455	0.30848
2	0.85987	0.94566	0.05557	0.13944
3	-1.22845	-1.11687	0.17583	0.25218
4	0.70728	0.54800	0.21707	0.07894
5	-2.53325	-2.69609	-0.45358	1.33326

Table S23: Transition Dipole Electric Moments and oscillator strength (f) of first 5 excited states for (P)-enantiomer.

State	X component	Y component	Z component	f
1	0.1212	2.11669	0.04392	0.30946
2	1.22791	-0.28100	-0.03486	0.13519
3	-1.61492	0.21061	0.29957	0.24812
4	1.09924	-0.08734	0.15084	0.11517
5	3.60363	-0.75831	0.18075	1.30492

Table S24: Transition Dipole Magnetic Moments of first 5 excited states for (P)-enantiomer.

State	X component	Y component	Z component
1	0.18208<br>(-0.18208)	-0.06253<br>(0.06255)	-0.37579<br>(0.37573)
2	0.07509<br>(0.07506)	-0.20103<br>(0.20109)	-0.00540<br>(0.00545)
3	0.11170<br>(-0.11176)	0.33131<br>(-0.33143)	0.29865<br>(-0.29946)
4	-0.01037<br>(0.01039)	0.08073<br>(-0.08085)	0.09046<br>(-0.09065)
5	-0.16873<br>(-0.16873)	-0.78295<br>(-0.78325)	0.03827<br>(0.03875)

References

(1) Bedi, A.; Manor Armon, A.; Diskin-Posner, Y.; Bogosalvsky, B.; Gidron, O. Controlling the Helicity of π-Conjugated Oligomers by Tuning the Aromatic Backbone Twist. *Nat Commun* **2022**, *13* (1), 451. https://doi.org/10.1038/s41467-022-28072-7.

(2) O'Driscoll, L. J.; Wang, X.; Jay, M.; Batsanov, A. S.; Sadeghi, H.; Lambert, C. J.; Robinson, B. J.; Bryce, M. R. Carbazole-Based Tetrapodal Anchor Groups for Gold Surfaces: Synthesis and Conductance Properties. *Angewandte Chemie International Edition* **2020**, *59* (2), 882–889. https://doi.org/10.1002/anie.201911652.
